# A Hypoxia‐Responsive Single‐Atom Sonozyme for Targeted Sonocatalytic Therapy in Alleviating Atherosclerotic Plaque

**DOI:** 10.1002/advs.202505058

**Published:** 2025-09-23

**Authors:** Qiaofei Chen, Guotao Yuan, Zhiwen Liu, Zhengyu Cao, Li He, Ruonan Li, Tongsheng Huang, Minglong Zheng, Kexin Wen, Canxia Huang, Shuang Zhu, Pingyu Zhang, Jingfeng Wang, Yuling Zhang, Yue Pan

**Affiliations:** ^1^ Department of Cardiology Guangdong Provincial Key Laboratory of Malignant Tumor Epigenetics and Gene Regulation Guangdong‐Hong Kong Joint Laboratory for RNA Medicine Medical Research Center Sun Yat‐sen Memorial Hospital Sun Yat‐sen University Guangzhou 510120 China; ^2^ School of Life Sciences and Biopharmaceutics Guangdong Pharmaceutical University Guangzhou 510006 China; ^3^ College of Chemistry and Environmental Engineering Shenzhen University Shenzhen 518037 China; ^4^ Department of Otolaryngology Longgang E.N.T. Hospital & Shenzhen Key Laboratory of E.N.T. Shenzhen 518116 China

**Keywords:** atherosclerosis, plaque microenvironment, single‐atom catalyst, single‐atom nanozymes, sonocatalytic therapy

## Abstract

Sonocatalytic therapy (SCT) offers a non‐invasive and deep tissue‐penetrating approach to addressing the pathological challenges of atherosclerosis. However, its therapeutic efficacy remains limited by the lack of efficient sonosensitizers. A critical challenge in SCT is simultaneously leveraging beneficial plaque microenvironment factors, such as elevated H_2_O_2_ levels, while mitigating adverse conditions, including hypoxia. Herein, a microenvironment‐regulatable single‐atom sonozyme system is presented to enable effective SCT while simultaneously refining the lesion microenvironment. The single‐atom manganese catalyst (SMC) is synthesized via MOF‐derived precursor pyrolysis followed by ion implantation, yielding atomically precise four‐coordinated active sites. Functionalized with hyaluronic acid (HA) facilitates targeted delivery of SMC‐HA to M1 macrophages. Under ultrasound (US), SMC‐HA effectively eliminates M1 macrophages, thereby reducing plaque burden and promoting lesion regression in two *ApoE^−/−^* mice models. Overall, SMC‐HA reinforces its role as an advanced sonosensitizer for SCT. This study establishes SMC‐HA‐mediated SCT as a promising therapeutic strategy for atherosclerotic plaque treatment.

## Graphical abstract

A tetranitrogen‐coordinated single‐atom manganese catalyst (SMC) is fabricated with outstanding sonosensitization efficiency and multi‐enzyme‐like catalytic properties. To enable better targeting of M1 macrophages, hyaluronic acid (HA) is applied to modify the surface of SMC, yielding SMC‐HA nanozymes. The SMC‐HA exhibits superior sonocatalytic activity. Finally, the as‐synthesized SMC‐HA demonstrates significantly enhanced anti‐atherosclerotic effect in *ApoE^–/–^* mouse model.

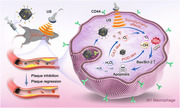

## 1 Introduction

Cardiovascular diseases (CVDs) remain the leading cause of mortality worldwide, with atherosclerotic plaque rupture being a major trigger for acute cardiovascular events including myocardial infraction and stroke.^[^ ^1^, ^2^, ^3^ ^]^ Current therapeutic strategies for atherosclerosis (AS) primarily include pharmacological interventions and surgical procedures. However, pharmacological approaches, such as long‐term statin therapy suffer from poor patient compliance and potential side effects.^[^ ^4^, ^5^ ^]^ Surgical procedures like stent implantation and endarterectomy are inherently invasive and pose risks such as intravascular restenosis.^[^ ^6^, ^7^ ^]^ Consequently, there is an urgent need to develop a non‐invasive, efficient, and targeted therapy for atherosclerotic plaques. The emergence of nanotechnology has brought a dawn to the diagnosis and treatment of AS.^[^ ^8^, ^9^, ^10^ ^]^

Given the pivotal role of macrophages in the progression of AS, macrophage‐targeted nanomedicine has gained increasing attention for AS diagnosis and treatment.^[^ ^11^, ^12^ ^]^ Functionally, macrophages can be categorized into pro‐inflammatory M1 and anti‐inflammatory M2 subtypes. M1 macrophages secrete high levels of inflammatory cytokines, maintaining a pro‐inflammatory microenvironment that promotes plaque destabilization. Therefore, the selective targeting and elimination of M1 macrophages has emerged as a promising therapeutic strategy for reducing inflammation and enhancing plaque stability.

Sonocatalytic therapy (SCT) is an emerging nanotechnology that utilizes localized ultrasound (US) to activate sonosensitizers, offering advantages such as non‐invasiveness, deep tissue penetration, and operational simplicity.^[^ ^13^, ^14^, ^15^ ^]^ In our previous study, we successfully developed a high‐performance SCT platform based on hyaluronic acid (HA) and PEG modified CuS/TiO_2_ nanosheets (HA‐HNSs), which exhibited exceptional potential for the treatment of early‐stage AS.^[^ ^16^ ^]^ Despite its promise, most current SCT approaches rely on conventional organic sonosensitizers, such as protoporphyrin IX, sinoporphyrin sodium, and curcumin.^[^ ^17^, ^18^, ^19^ ^]^ These compounds suffer from low chemical stability, poor water solubility, and suboptimal therapeutic efficiency. Although inorganic sonosensitizers have been extensively investigated in preclinical cancer models, they are often hindered by poor biodegradability and potential biosafety concerns. Moreover, their application in AS remains insufficiently explored. These limitations underscore the pressing need for innovative sonosensitizers that can achieve high sonodynamic efficiency while maintaining low metal content, thereby improving the therapeutic performance of SCT in AS. In this context, single‐atom catalysts (SACs) have emerged as a novel class of nanomaterials featuring atomically dispersed active sites and tunable coordination environments.^[^ ^20^ ^]^ SACs not only maximize catalytic activity and metal atom utilization but also reduce potential metal‐associated toxicity. Their well‐defined catalytic behavior provides a promising strategy to address the dual challenges of inefficient sonosensitization and excessive metal burden, paving the way for next‐generation SCT agents.

Another major challenge in SCT‐based AS therapy lies in the hypoxic microenvironment within atherosclerotic plaques. Hypoxia not only aggravates inflammation and elevates the risk of plaque rupture but also compromises the efficacy of SCT.^[^ ^21^ ^]^ The excessive endogenous hydrogen peroxide (H_2_O_2_) accumulation under hypoxic conditions often leads to cellular adaptation rather than apoptosis.^[^ ^22^ ^]^ Therefore, converting H_2_O_2_ into highly cytotoxic singlet oxygen (^1^O_2_) and hydroxyl radicals (·OH) represents a critical strategy to exceed cellular antioxidant defenses, thereby triggering apoptosis pathways.^[^ ^23^ ^]^ For instance, titanium monoxide (TiO_1+x_ nanozymes have demonstrated efficacy in improving the tumor microenvironment by catalyzing endogenous H_2_O_2_ into ·OH for enhanced therapy.^[^ ^24^ ^]^ However, the potential application of nanozymes in AS treatment remains largely uninvestigated.

**Scheme 1:**
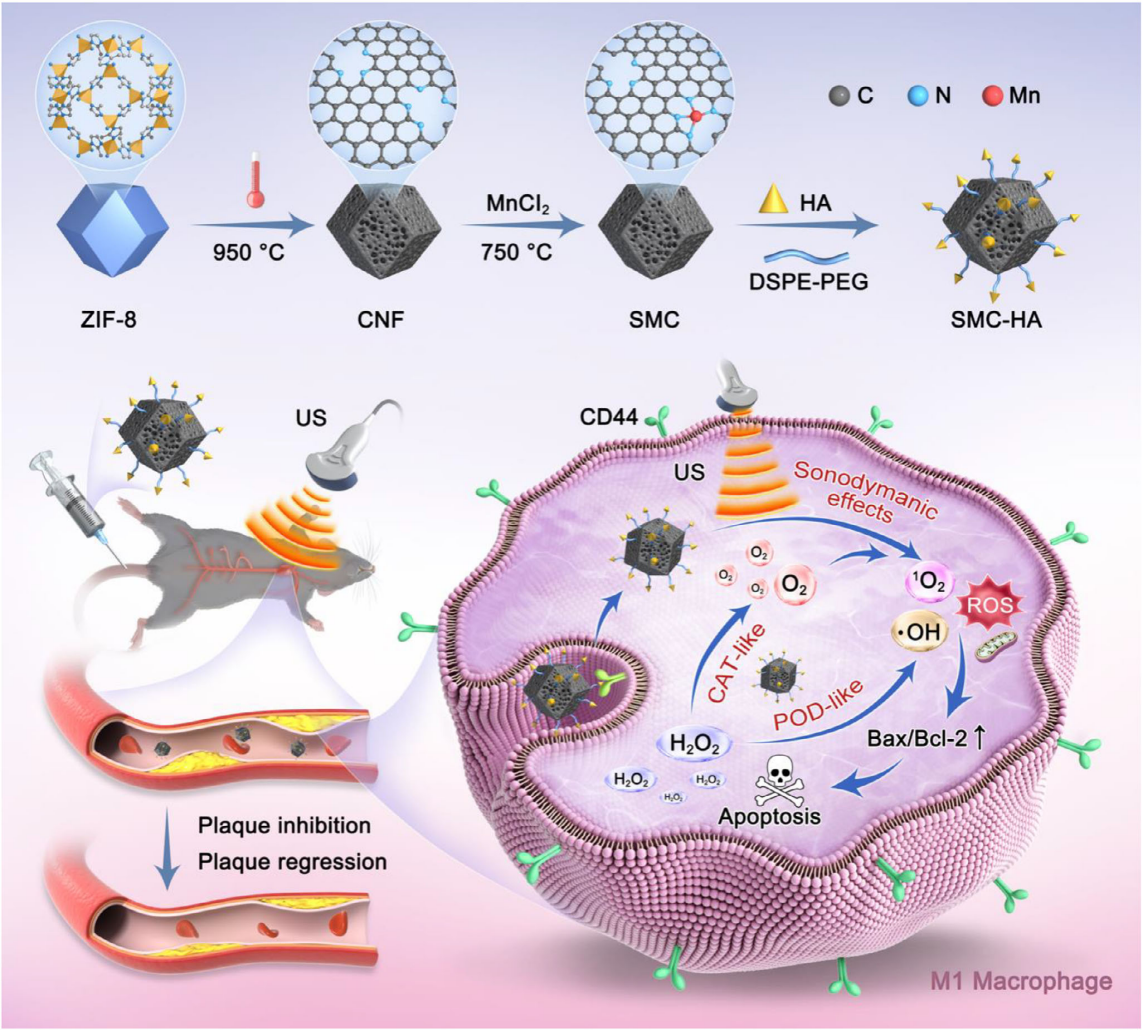
Schematic illustration of the synthesis procedure for SMC‐HA and its application in SCT targeting plaque treatment.

In this study, the microenvironment‐regulatable design of a single‐atom sonozyme system was performed (**Scheme** **1**). Specifically, we synthesized a tetranitrogen‐coordinated single‐atom manganese catalyst (SMC) using a carbon restructuring strategy to achieve high sonosensitization efficiency and multi‐enzyme‐like catalytic activity. To enable selective M1 macrophage targeting, HA was applied to modify the surface of SMC, yielding SMC‐HA nanozymes. Our findings demonstrated that SMC‐HA, in combination with US irradiation, significantly enhanced reactive oxygen species (ROS) levels, triggered apoptosis of M1 macrophages. In two *ApoE^−/−^* mouse models, SMC‐HA‐mediated SCT effectively inhibited plaque progression, promoted plaque regression, and enhanced plaque stability. This integrated approach provides a promising strategy for SCT‐based AS therapy by simultaneously modulating the plaque microenvironment and ensuring high therapeutic efficacy with favorable biosafety.

## 2 Results and Discussion

### 2.1 Characterization of SMC

**Figure 1:**
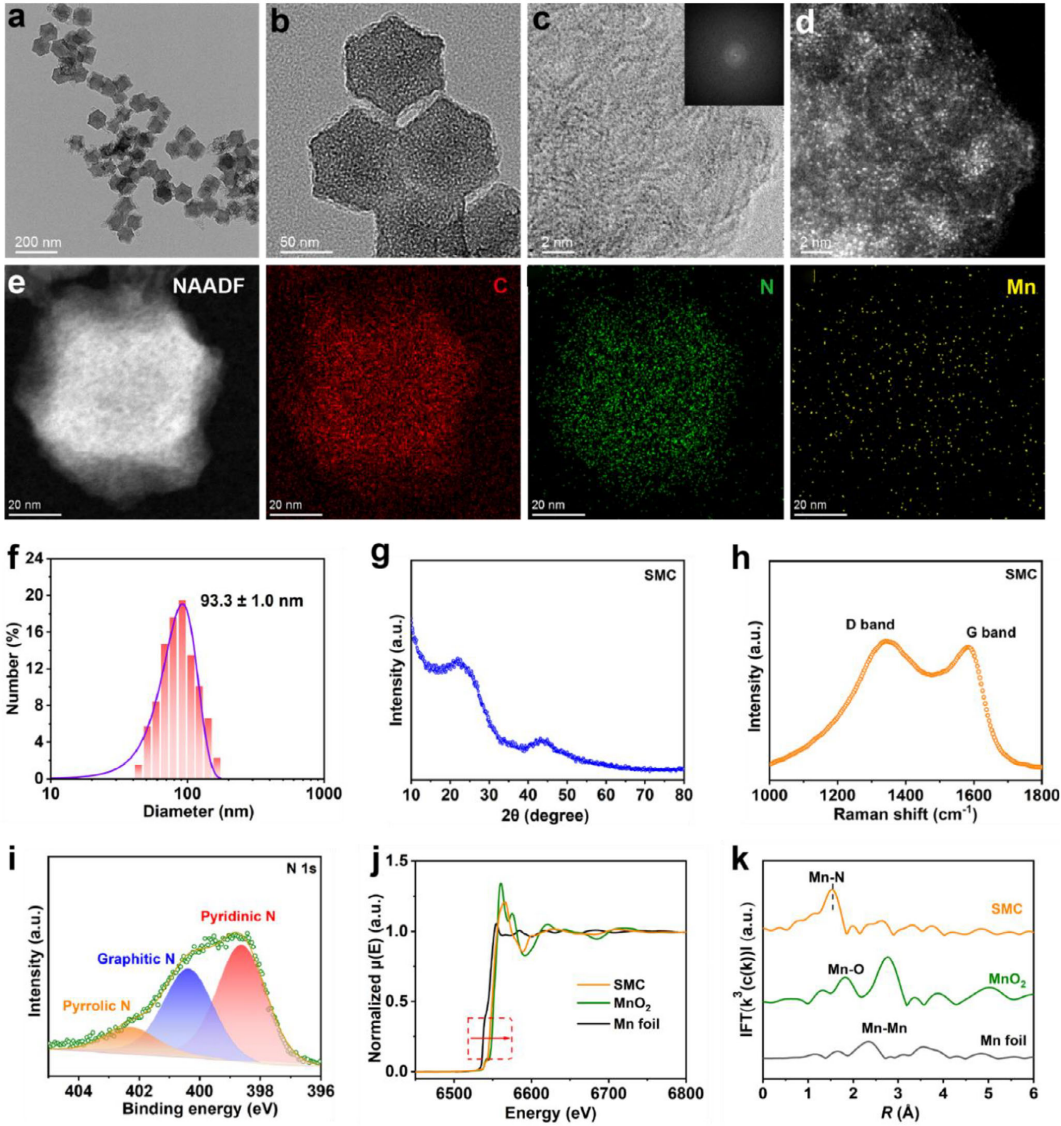
Characterization of SMC. a,b) TEM images of SMC. c,d) Aberration‐corrected HAADF‐STEM images of SMC. e) HAADF‐STEM micrographs and the corresponding EDX elemental maps of SMC. f) DLS analysis of SMC. g) XRD spectrum of SMC. (h) Raman spectrum of SMC. i) N 1s XPS spectrum of SMC. j) XANES spectrum of SMC. k) Fourier‐transformed EXAFS of SMC.

Transmission electron microscopy (TEM) analysis of SMC revealed a porous polyhedral carbon structure with an average diameter of 93.3 ± 1.0 nm (**Figure** **1**a,b). High‐angle annular dark‐field scanning transmission electron microscopy (HAADF‐STEM) images displayed numerous bright spots dispersed on the carbon framework, indicative of atomically distributed manganese sites (Figure 1c,d). Elemental mapping further verified the homogeneous distribution of carbon (C), nitrogen (N), and manganese (Mn) throughout the SMC matrix (Figure 1e). Dynamic light scattering (DLS) analysis confirmed a hydrodynamic diameter of 93.3 ± 1.0 nm (Figure 1f), consistent with the TEM observations. X‐ray diffraction (XRD) patterns exhibited two broad peaks at 23.5° and 42.6° (Figure 1g), corresponding to the (002) and (101) planes of graphitic carbon, indicating the absence of metallic Mn. Raman spectroscopy identified characteristic D and G bands at 1331.3 and 1583.0 cm^−1^, which are attributed to sp3 dangling carbon and in‐plane sp2 carbon, respectively (Figure 1h). X‐ray photoelectron spectroscopy (XPS) of the N 1s spectrum highlighted three distinct nitrogen species: pyridinic N (398.6 eV), pyrrolic N (401.2 eV), and graphitic N (402.3 eV) (Figure 1i). According to the X‐ray absorption near edge structure (XANES) analysis, Mn in SMC primarily existed in oxidation states ranging from +2 to +3 (Figure 1j). Fourier‐transformed extended X‐ray absorption fine structure (EXAFS) spectra displayed no detectable Mn─Mn bonding peak, whereas a distinct peak at 1.66 Å corresponded to Mn─N coordination (Figure 1k). Collectively, these results confirm the successful synthesis of a tetranitrogen‐coordinated single‐atom manganese catalyst.

To systematically investigate the long‐term stability of our catalyst, we first monitored the nanomaterial's hydrodynamic size evolution in various physiological solutions (PBS, DMEM, and FBS) over 8 days. Notably, SMC exhibited excellent stability without observable sedimentation (Figure S1a, Supporting Information) or significant alterations in particle size distribution (Figure S1b, Supporting Information). To enhance biocompatibility and achieve selective targeting of M1 macrophages, SMC was coated with HA and SPE‐PEG. Fourier transform infrared (FT‐IR) spectroscopy (Figure S2, Supporting Information) revealed distinct absorption peaks corresponding to PEG, including the saturated C─H stretching vibrations typically observed between 2800 and 3000 cm^−1^. For SMC‐HA, additional characteristic peaks of HA were detected, including strong O─H and carboxyl group absorptions, confirming the successful conjugation of HA to SMC‐PEG. The O─H stretching vibrations appeared in the 3650–3200 cm^−1^ range, while the carboxyl stretching peak was prominently observed between 1700 and 1730 cm^−1^, further validating the synthesis of both SMC‐PEG and SMC‐HA. Moreover, both SMC‐PEG and SMC‐HA exhibited negative zeta potentials (Figure S3, Supporting Information). Notably, the hydrodynamic size of SMC‐HA (Figure S4, Supporting Information) was larger than that of unmodified SMC, confirming the successful surface modification.

### 2.2 Sonocatalytic Performance and Enzyme‐Like Activity of SMC

**Figure 2:**
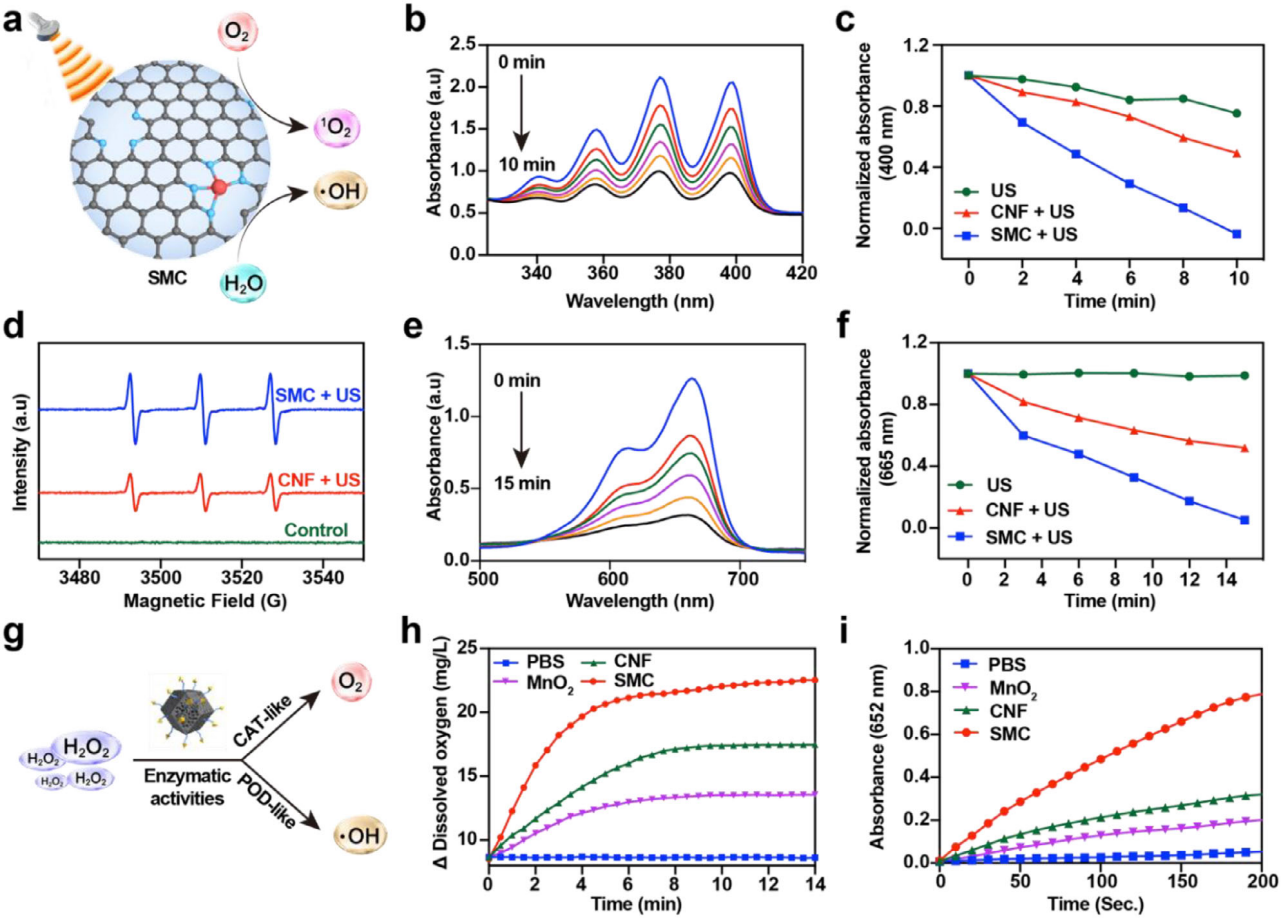
Sonocatalytic performance, CAT‐like, and POD‐like activities of SMC. a) Schematic illustration of the mechanism of SMC‐mediated SCT procedure. b) The absorption of ABDA in the SMC + US group (1.0 W cm^−2^ 1.0 MHz, 50% duty). c) The absorption intensities at 400 nm of ABDA following treatment with PBS, CNF, and SMC followed by US irradiation for 10 min. d) ESR spectra illustrating the generation of ^1^O_2_ by the samples under US irradiation. e) The absorbance of MB in the SMC + US group. f) The absorption intensities at 665 nm of MB following treatment with PBS, CNF, and SMC followed by US irradiation for 15 min. g) Schematic diagram of the reactive process of the CAT‐like and POD‐like activities of SMC. h) CAT‐like activity of CNF, MnO_2_, and SMC. i) POD‐like activity of CNF, MnO_2_, and SMC.

The therapeutic efficacy of sonosensitizers largely depends on their capacity to generate ^1^O_2_ and ·OH under US irradiation (**Figure** **2**a). To assess ^1^O_2_ production, the optical density changes of PBS, carbon nanoframe (CNF), and SMC were monitored using 9,10‐Anthracenediyl‐bis (methylene) dimalonic acid (ABDA) as a probe. After 10 min of US exposure (1.0 W cm^−2^, 1.0 MHz, 50% duty), a substantial decrease in the absorption peak was observed in the SMC group (Figure 2b), whereas only minor changes occurred in the PBS and CNF groups (Figure S5a,b, Supporting Information). Quantitative absorbance measurements at 400 nm further confirmed the most pronounced reduction in the SMC group (Figure 2c). Additionally, electron spin resonance (ESR) spectroscopy demonstrated that SMC generated the strongest 1:1:1 characteristic signal for ^1^O_2_ under US irradiation (Figure 2d). To further evaluate ⋅OH production, methylene blue (MB) was employed as a detection probe. As shown in Figure 2e and Figure S6 (Supporting Information), SMC caused a more substantial decrease in the absorption peak than CNF. Quantitative analysis at 665 nm confirmed that SMC produced significantly higher levels of ⋅OH than CNF following 15 min of US exposure (Figure 2f). We further employed TMB as a probe to effectively evaluate the generation of hydroxyl radicals. AS shown in Figure S7 (Supporting Information), the results demonstrate significant •OH production, which is consistent with our previous findings using the MB probe. These findings suggest that Mn atom incorporation markedly enhances the sonocatalytic performance of SMC, supporting its robust ROS‐mediated therapeutic capacity.

Elevated H_2_O_2_ levels within atherosclerotic plaques serve as abundant substrates for enzymatic reactions catalyzed by catalase (CAT) and peroxidase (POD). SMC exhibited dual enzyme‐mimetic activities by decomposing H_2_O_2_ into molecular O_2_ via CAT‐like behavior and converting H_2_O_2_ into ·OH through POD‐like catalysis (Figure 2g). To assess its CAT‐like function, we measured the O_2_ generation capabilities of SMC, CNF, and MnO_2_ in H_2_O_2_ solutions using a dissolved oxygen meter. As shown in Figure 2h, SMC significantly increased the dissolved O_2_ concentration from 8.5 to 22.59 mg L^−1^ within 14 min, outperforming both MnO_2_ and CNF. Additionally, the O_2_ concentration in SMC‐containing solutions increased proportionally with higher H_2_O_2_ concentrations (Figure S8, Supporting Information), and visible O_2_ bubbles adhered to the inner tube walls were more and bigger (Figure S9, Supporting Information). To further confirm the POD‐like activity of SMC, 3,3′,5,5′‐tetramethylbenzidine (TMB), a chromogenic substrate, was employed as an indicator. Upon oxidation, TMB formed oxTMB with a characteristic absorption peak at 652 nm. As illustrated in Figure 2i, SMC exhibited a significantly greater and more rapid increase in the oxTMB absorption peak compared to CNF and MnO_2_. Furthermore, the magnitude of this increase positively correlated with the concentration of H_2_O_2_ and SMC (Figures S10 and S11, Supporting Information). In parallel, we compared MB degradation under ultrasound irradiation among PBS, CNF, and SMC. The SMC group exhibited significantly higher MB removal efficiency than the control groups (PBS and CNF), with the degradation effect enhancing progressively with increasing H_2_O_2_ and SMC concentrations (Figure S12, Supporting Information). pH‐ and H_2_O_2_‐dependent catalytic characterization of SMC was systematically investigated through nanozyme activity assays. As shown in Figure S13 (Supporting Information), the POD‐like activity of SMC exhibits a strong pH dependence, showing a dramatic increase with decreasing pH and reaching maximum activity at pH 3.0. In striking contrast, CAT‐like activity displays optimal performance under neutral (pH 7.0) and alkaline (pH 9.0) conditions, with significantly reduced activity in acidic environments. These results provide compelling evidence for the pH‐dependent dual‐enzyme mimicry of SMC: functioning as an efficient POD mimic in acidic conditions while switching to CAT‐like behavior at physiological pH.

### 2.3 In Vitro SMC‐HA‐Mediated SCT Effects

**Figure 3:**
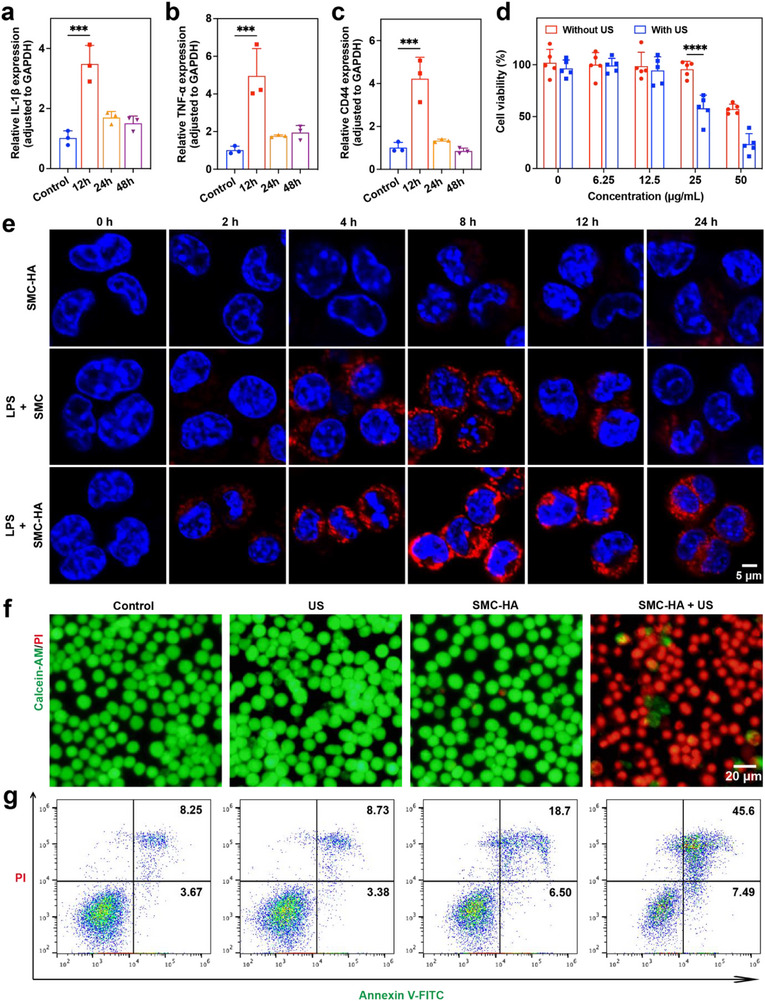
In Vitro SMC‐HA‐mediated SCT effects. a–c) Quantitative RT‐PCR analysis of RNA levels of a) IL‐1β, b) TNF‐α, and c) CD44 in RAW264.7 cells following LPS stimulation at different time points (*n* = 3). d) Cell viability of M1 macrophages incubated with SMC‐HA at various concentrations with/without US irradiation (*n* = 5). e) Representative confocal fluorescence images of RAW 264.7 macrophages incubated with PBS, LPS + SMC, and LPS + SMC‐HA. Scale bar: 5 µm. f) Live/dead staining images of M1 macrophages with various treatments. Scale bar: 20 µm. g) Flow cytometry analysis of M1 macrophages with various treatments. Data are presented as mean values ± SD. ***p < 0.001, ****p < 0.0001.

Macrophages are broadly classified into pro‐inflammatory M1 and anti‐inflammatory M2 phenotypes, with M1 macrophages constituting the predominant pathological subset in unstable atherosclerotic plaques.^[^ ^25^ ^]^ In this study, lipopolysaccharide (LPS, 1 µg mL^−1^) was used to induce the polarization of RAW264.7 macrophages toward the M1 phenotype.^[^ ^26^, ^27^ ^]^ After a 12 h incubation, RAW264.7 macrophages exhibited maximal secretion of inflammatory cytokines, including interleukin‐1β (IL‐1β) (**Figure** **3**a) and tumor necrosis factor‐α (TNF‐α) (Figure 3b). Additionally, CD44 expression in LPS‐treated macrophages was elevated by 4.23‐fold relative to the untreated control group (Figure 3c). Subsequently, the cytotoxicity of SMC‐HA, with and without US irradiation, was evaluated using the cell counting kit‐8 (CCK‐8) assay. No significant reduction in cell viability was observed at concentrations up to 25 µg mL^−1^. However, under US exposure, a dramatic decrease in cell viability was noted when SMC‐HA concentrations exceeded 12.5 µg mL^−1^ (Figure 3d). Additionally, cell viability declined progressively with increasing SMC‐HA concentrations and higher US intensities (Figure S14, Supporting Information). Furthermore, US stimulation significantly reduced viability across all BMDM subtypes (M0, M1, and M2), with the inhibitory effects exhibiting concentration dependence (Figure S15, Supporting Information).

To assess the targeting ability of SMC‐HA toward M1 macrophages, Cy5.5‐labeled nanoparticles were utilized, and intracellular fluorescence intensity was analyzed using confocal laser scanning microscopy (CLSM). The results showed that LPS‐induced macrophages internalized significantly more SMC‐HA compared to SMC, with peak uptake observed at 8 h post‐incubation (Figure 3e; Figure S16, Supporting Information). In contrast, weak fluorescence was detected in untreated RAW264.7 cells despite exposure to the same concentration of SMC‐HA. This diminished signal is likely attributable to the low surface expression of CD44, resulting in fewer binding sites. Following the steps shown in Figure S17 (Supporting Information), we extended our analysis to include additional cell lines such as bone marrow‐derived macrophages (BMDMs). As validated by qRT‐PCR analysis (Figure S18, Supporting Information), the BMDMs were successfully polarized into classically activated (M1, via LPS) and alternatively activated (M2, via IL‐4) phenotypes. Uptake studies demonstrated that M1 macrophages showed preferential internalization of SMC‐HA‐Cy5.5 compared to their M0 and M2 counterparts (Figure S19, Supporting Information), with this selective uptake being effectively blocked by free HA pretreatment (Figure S20, Supporting Information), thereby confirming CD44‐mediated targeting of pro‐inflammatory macrophages by our HA‐functionalized nanomaterial. These findings demonstrate that HA modification enhances the CD44‐mediated targeting specificity of SMC‐HA toward M1 macrophages.

Live/dead staining verified that the combined treatment of SMC‐HA and US effectively induced cell death, whereas US exposure alone exerted no detectable cytotoxic effect (Figure 3f). Flow cytometry analysis demonstrated that this cytotoxicity was predominantly driven by apoptosis, with an apoptotic rate of ≈52.46% observed in the SMC‐HA + US group (Figure 3g; Figure S21, Supporting Information). This high rate of apoptosis highlights the strong efficacy of SMC‐HA in selectively inducing M1 macrophage death upon US irradiation.

### 2.4 The Mechanism of SMC‐HA‐Mediated Apoptosis of M1 Macrophages

**Figure 4:**
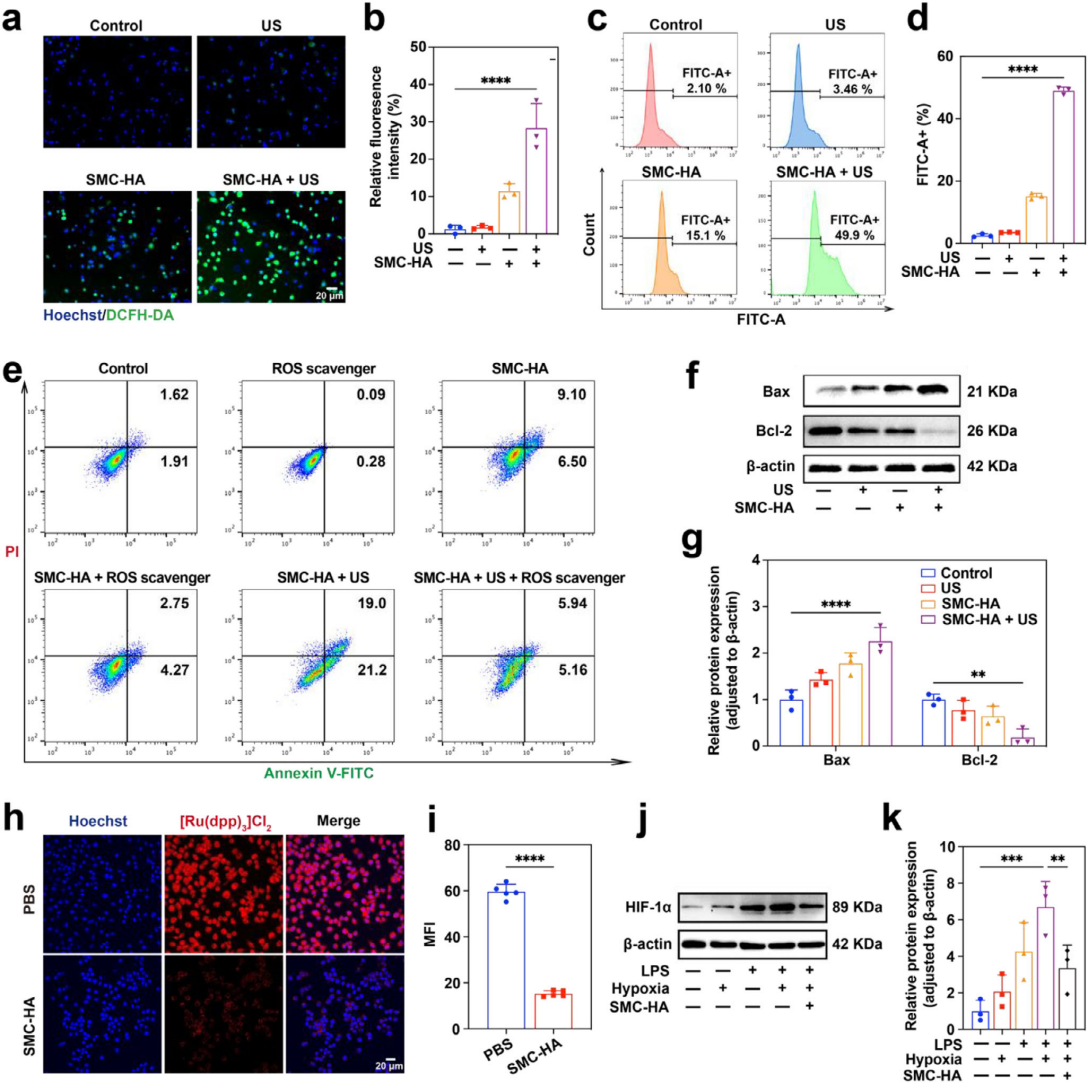
In vitro analysis of ROS‐generation capability, and hypoxia alleviation after SMC‐HA‐mediated SCT. a) Representative fluorescence microscopy images of the intracellular ROS generation after different treatments. Scale bar: 20 µm. b) Quantitative analysis of the fluorescent intensity in (a) (*n* = 3). c) Flow cytometry analysis of FITC‐A+ cells after different treatments. d) Quantitative analysis of the FITC‐A+ ratio in (b) (*n* = 3). e) Flow cytometry analysis of M1 macrophages with various treatments. f) Western blot bands of Bax and Bcl‐2. g) Quantitative analysis of the expression of Bax and Bcl‐2 in different groups (*n* = 3). h) SMC‐HA‐induced intracellular oxygenation in M1 macrophages as examined using the oxygen indicator [Ru(dpp)_3_]Cl_2_. Scale bar: 20 µm. i) Quantitative analysis of the fluorescent intensity in (d) (*n* = 3). j) Western blot bands of HIF‐1α. k) Quantitative analysis of the expression of HIF‐1α. Data are presented as mean values ± SD. **p < 0.01, ***p < 0.001, ****p < 0.0001.

ROS generation constitutes the pivotal event in inertial cavitation, the widely recognized primary mechanism of SCT. To clarify the correlation between SCT‐induced M1 macrophage apoptosis and ROS production, we evaluated intracellular ROS levels using the fluorescent DCFH‐DA probe. Fluorescence microscopy revealed that US irradiation alone did not significantly alter intracellular ROS levels. Compared with the control group, the SMC‐HA‐treated cells exhibited weak green fluorescence signals (**Figure** **4**a,b). In contrast, cells treated with the combination of SMC‐HA and US displayed markedly enhanced fluorescence intensity, a finding that was further corroborated by flow cytometry analysis (Figure 4c,d).

To help clarify the causal relationships and establish the causative link between ROS generation and cellular apoptosis, we performed ROS scavenger intervention experiments. As shown in Figure 4e and Figure S22 (Supporting Information), SMC‐HA treatment alone induced moderate apoptosis (14.43%), which was reduced by 30.1% (to 10.08%) upon ROS scavenger co‐treatment. Strikingly, the combination of SMC‐HA with US irradiation dramatically enhanced apoptosis to 40.67% compared to SMC‐HA alone, confirming robust sonosensitization. However, this pro‐apoptotic effect was almost completely abolished by ROS scavenger, with apoptosis plummeting to 10.08%, a 4.03‐fold reduction. These findings indicate that SMC‐HA + US promotes macrophage apoptosis via ROS‐driven mitochondrial dysfunction, highlighting the critical role of oxidative stress in this therapeutic mechanism.

Endogenous ROS generation is primarily attributed to leakage from the mitochondrial electron transport chain.^[^ ^28^ ^]^ Given the pivotal role of Bcl‐2 family proteins in regulating mitochondrial‐mediated apoptosis, western blot analysis was conducted to assess the expression of Bax and Bcl‐2 under different treatment conditions. As shown in Figure 4f,g, SMC‐HA treatment upregulated Bax and downregulated Bcl‐2, with the most significant changes observed in the SMC‐HA + US group. These results indicate that SMC‐HA‐mediated SCT triggers apoptosis through the mitochondrial‐dependent intrinsic pathway.

### 2.5 In Vitro Performance in Alleviating Hypoxia

As atherosclerotic plaques progress, the arterial wall thickens beyond the oxygen diffusion limit (100–200 µm),^[^ ^29^ ^]^ while elevated metabolic activity further increases oxygen consumption, resulting in pronounced hypoxia in macrophages.^[^ ^30^ ^]^ This hypoxic microenvironment severely compromises the efficacy of SCT. Meanwhile, the elevated H_2_O_2_ levels in plaques may serve as substrates for catalase (CAT)‐like activity to generate oxygen. Therefore, the strong pro‐apoptotic effect observed with SMC‐HA‐mediated SCT in M1 macrophages may partially result from its CAT‐like enzymatic function. To test this hypothesis, oxygen‐sensitive [Ru(dpp)_3_]Cl_2_ was used to assess intracellular O_2_ generation. A significant decrease in the red fluorescence signal was observed in M1 macrophages treated with SMC‐HA, indicating that SMC‐HA generated substantial amounts of O_2_ within these cells (Figure 4h,i). Following the isolation protocol (Figure S15, Supporting Information), BMDMs were successfully polarized into distinct functional phenotypes for systematic evaluation of SMC‐HA's hypoxia‐modulating effects. [Ru(dpp)_3_]Cl_2_ fluorescence imaging revealed minimal baseline signal across all phenotypes under normoxic conditions, with no significant changes following SMC‐HA treatment, confirming maintained oxygen homeostasis. Strikingly, hypoxia‐induced fluorescence intensification was consistently observed in all macrophage subtypes, while subsequent SMC‐HA administration produced significant signal attenuation (Figure S23, Supporting Information). This oxygen‐dependent fluorescence quenching demonstrates SMC‐HA's capacity to alleviate intracellular hypoxia.

Hypoxia‐inducible factor‐1α (HIF‐1α) is a central transcriptional regulator of cellular adaptation to hypoxia and is reportedly overexpressed in macrophages within atherosclerotic lesions.^[^ ^31^ ^]^ However, it remains unclear whether alleviating cellular hypoxia would affect HIF‐1α expression. To explore this, LPS‐treated macrophages were cultured under hypoxic conditions. LPS stimulation significantly upregulated HIF‐1α protein expression in macrophages to 4.26‐fold of the control level, consistent with previous reports by Evanna et al.^[^ ^32^ ^]^ When combined with hypoxic culture, HIF‐1α levels further increased to 6.70‐fold, representing a 57.3% increase compared to LPS treatment alone. Notably, SMC‐HA intervention effectively reduced HIF‐1α expression to 3.36‐fold of control, representing a 49.8% decrease compared to the LPS + hypoxia group (Figure 4j,k). These findings suggest that SMC‐HA alleviates cellular hypoxia and consequently downregulates HIF‐1α expression in M1 macrophages.

### 2.6 SMC‐HA Targeted M1 Macrophages in Atherosclerotic Plaques

**Figure 5:**
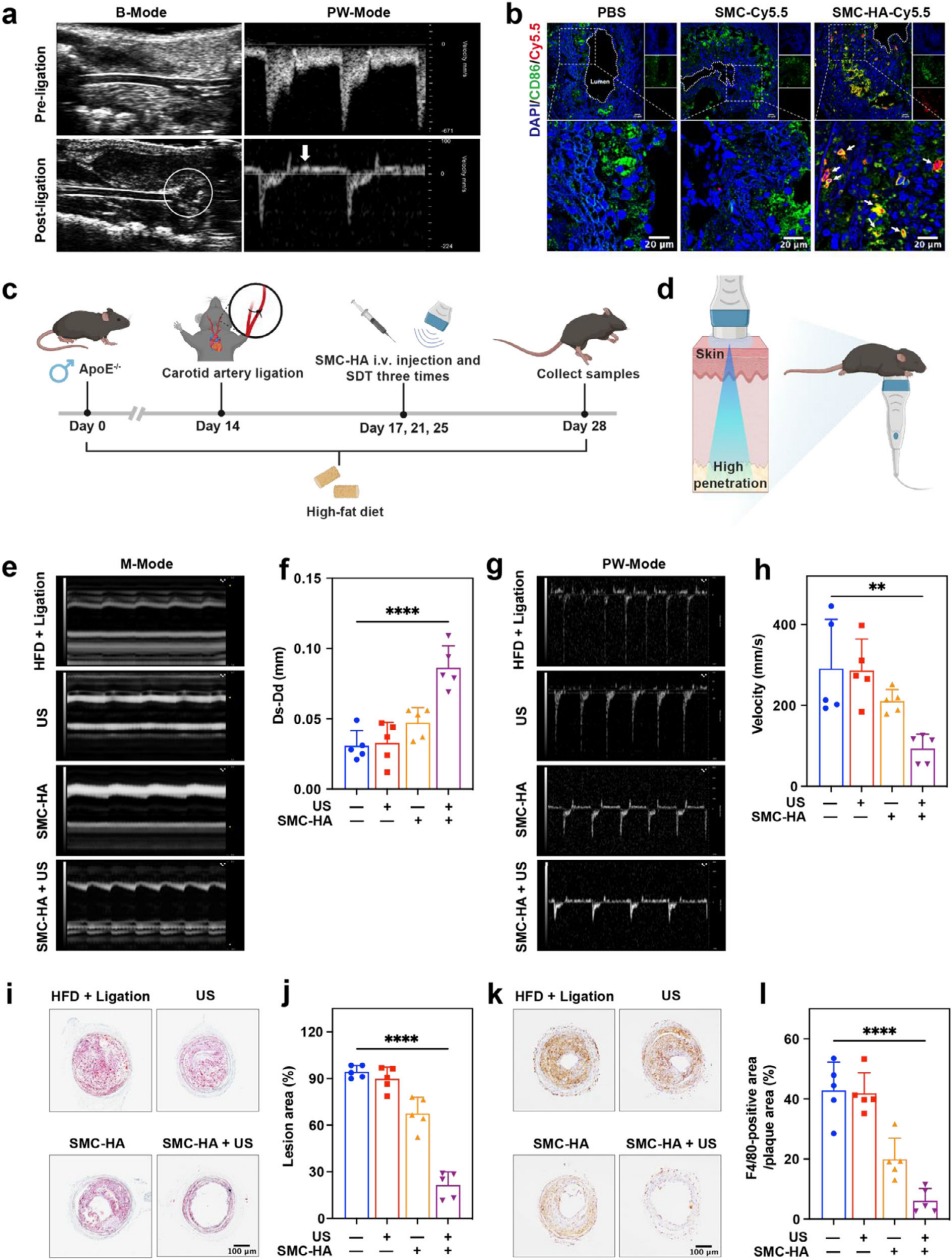
SMC‐HA‐mediated SCT shows preventive effects on AS in *ApoE* ^−/−^ mice with partial left carotid artery ligation and 4‐week high‐fat diet feeding. a) Representative B‐mode ultrasound images and flow velocity profiles of the LCCA (long axis). b) Representative confocal fluorescence images of LCCA transverse sections. Scale bar: 20 µm. c) Schematic representation of the in vivo experimental protocol in the carotid atherosclerotic plaque mice. d) The SCT process the carotid atherosclerotic plaque mice. e) M‐mode micro‐ultrasound measured LCCA pulsatility. f) The calculation and statistical analysis of the elastic diameter in (e) (*n* = 5). g) PW‐mode micro‐ultrasound measured blood flow velocity. h) The quantification of ultrasound‐derived maximal systolic velocity (V_max_) in (g) (*n* = 5). i) Representative microscopy images of LCCA transverse sections stained with Oil red O. Scale bar: 100 µm. j) Proportions of plaque area in (i) (*n* = 5). k) Representative microscopy images of LCCA transverse sections stained with F4/80. Scale bar: 100 µm. l) Proportions of F4/80‐positive area in (k) (*n* = 5). Data are presented as mean values ± SD. **p < 0.01, ****p < 0.0001. Schematic diagram created in https://BioRender.com.

Subsequently, we investigated the dominant cells targeted by SMC and SMC‐HA during plaque formation. Two AS models were established: one by performing partial carotid artery ligation combined with a 4‐week high‐fat diet (HFD) in *ApoE^−/−^* mice (Figures S24 and S25, Supporting Information), and the other by feeding *ApoE^−/−^* mice an HFD for 14 weeks (Figure S26, Supporting Information). To confirm the success of the ligation, the morphology and blood flow velocity of the left common carotid artery (LCCA) before and after ligation were assessed using high‐resolution ultrasonography. The results indicated that the branch of the LCCA was successfully blocked following ligation, accompanied by a decrease in systolic flow velocity and reversal of the flow direction during diastole (**Figure** **5**a).

Next, *ApoE^−/−^* mice 2 weeks after ligation combined with HFD received tail vein injections of either SMC‐Cy5.5 or SMC‐HA‐Cy5.5 (10 mg kg^−1^). The LCCA and aorta were harvested 24 h later. In vitro fluorescence imaging showed that SMC‐HA‐Cy5.5 exhibited a higher affinity for atherosclerotic plaques compared to PBS and SMC (Figures S27 and S28, Supporting Information). We further assessed the in vivo function of SMC‐HA‐Cy5.5. In vivo fluorescence imaging revealed that SMC‐HA‐Cy5.5 rapidly accumulated in the LCCA, with peak fluorescence observed at 24 h (Figure S29, Supporting Information). In contrast, weak fluorescence was observed in the LCCA of C57 mice fed a normal diet (Figure S30, Supporting Information), indicating that SMC‐HA‐Cy5.5 preferentially accumulated in the plaques of the LCCA. Additionally, SMC‐HA‐Cy5.5 was predominantly localized in M1 macrophages within the LCCA sections (Figure 5b). These findings demonstrate that SMC‐HA‐Cy5.5 specifically targets M1 macrophages in the plaques of both atherosclerosis models in mice.

### 2.7 SMC‐HA‐Mediated Sonocatalytic Therapy Alleviated Atherosclerosis Progression

To evaluate the therapeutic effect of SMC‐HA, SMC‐HA‐mediated SCT was applied to *ApoE^−/−^* mice during atherosclerosis formation (Figure 5c). SMC‐HA was administered intravenously at a dosage of 10 mg kg^−1^ through tail vein injection. As shown in Figure 5d, US (1.0 W cm^−2^ 10 min, 1.0 MHz, 50% duty cycle) was used to irradiate the neck of mice in the experimental groups receiving US irradiation. We observed that the plaque was easily visualized within the LCCA of *ApoE^−/−^* mice fed a HFD combined with ligation. Interestingly, SMC‐HA‐mediated SCT significantly reduced plaque formation (Figures S31 and S32, Supporting Information). In addition, we measured the difference between systolic diameter (Ds) and diastolic diameter (Dd) of the LCCA, which reflects vascular stiffness. The results revealed no significant changes in Ds‐Dd in the US group. However, this difference was significantly increased in the SMC‐HA + US group, indicating that SMC‐HA‐mediated SCT could reduce vascular stiffness in the LCCA (Figure 5e,f). Next, we measured the peak systolic maximum velocity (V_max_) and resistance index (RI) values of the LCCA under pulsed wave (PW) mode, which increased with the progression of AS. We observed that the V_max_ and RI values in the SMC‐HA + US group were significantly reduced compared to the HFD + Ligation group (Figure 5g,h; Figure S33, Supporting Information).

Additionally, oil red O staining revealed that plaques in the HFD + Ligation group and the US group occupied ≈94.32% and 89.78% of the total lumen area, respectively. Although plaque area decreased slightly in the SMC group, SMC‐HA‐mediated SCT significantly reduced the plaque area to ≈21.49% (Figure 5i,j). Furthermore, the macrophage count in plaques was significantly lower in the SMC‐HA + US group compared to the HFD + Ligation group (Figure 5k,l). These results indicated that micro‐ultrasound imaging offered a valuable non‐invasive approach for assessing the morphological and hemodynamic features of vulnerable plaques. Moreover, SMC‐HA‐mediated SCT effectively suppressed early atherosclerotic plaque formation in the LCCA of *ApoE^−/−^* mice fed with HFD combined with ligation by targeting and ablating macrophages. Immunofluorescence analysis of plaque HIF‐1α expression is exhibited in Figure S34 (Supporting Information). The result demonstrated the inherent oxygen‐generating capacity of SMC‐HA, leading to effective modulation of the hypoxic microenvironment within atherosclerotic lesions. The therapeutic efficacy was further enhanced by US activation, where sonocatalytically‐induced apoptosis of inflammatory macrophages mediated reduction of HIF‐1α signal intensity. The combination of hypoxia alleviation and targeted clearance of pro‐inflammatory macrophages establishes a novel therapeutic paradigm for combating hypoxia‐driven plaque progression while simultaneously addressing its inflammatory components.

**Figure 6:**
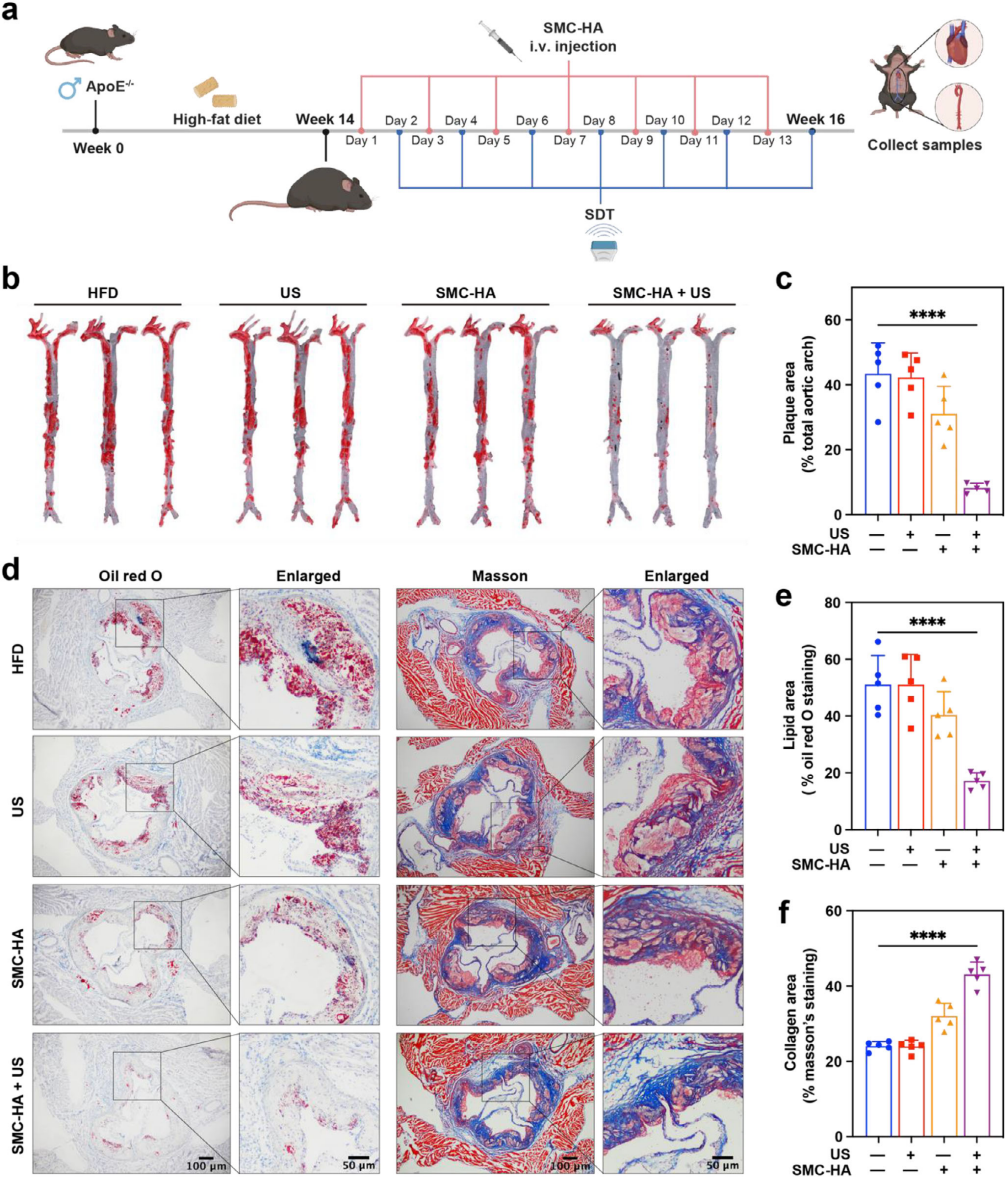
SMC‐HA‐mediated SCT shows therapeutic effects on AS in *ApoE* ^−/−^ mice with 14‐week high‐fat diet feeding. a) Schematic representation of the in vivo experimental protocol in the aortic atherosclerotic plaque mice. b,c) Representative images of en face aortas stained with ORO and quantified analysis of the Oil red O‐positive lesion area (*n* = 5). d) Representative images of aortic root stained with Oil red O and Masson. Scale bar of the left pictures: 100 µm. Scale bar of the right pictures below: 50 µm. e,f) Quantitative analyses of lipid and collagen areas shown in (d) (*n* = 5). Data are presented as mean values ± SD. ****p < 0.0001. Schematic diagram created in https://BioRender.com.

Next, the therapeutic effect of SMC‐HA‐mediated SCT on spontaneous AS formation in *ApoE^−/−^* mice was further assessed. The *ApoE^−/−^* mice were fed with long‐term HFD for 14 weeks, followed by SMC‐HA‐mediated SCT for an additional 2 weeks (**Figure** **6**a). Consistent with the previous treatment protocol, SMC‐HA (10 mg kg^−1^) was administered via tail vein injection, followed by US irradiation (1.0 W cm^−2^ 1.0 MHz, 50% duty cycle) after a 24 h incubation period. As observed in the aortas of *ApoE^−/−^* mice in the HFD group, plaques accounted for ≈43.41% of the total aorta, while plaques in the US group showed no significant change, indicating US alone could not reduce the plaque formation. Mice treated with SMC‐HA showed a reduction in plaque area to ≈31.05%, suggesting that the ROS generated by the peroxidase‐like catalytic activity of the SMC‐HA plays a role in plaque regression. However, when SMC‐HA was injected via tail vein and followed by additional US irradiation (1.0 W cm^−2^ 1.0 MHz, 50% duty cycle), the plaque area was further decreased to ≈8.22%, highlighting that the enzyme‐like activity synergized with its sonosensitizing ability, significantly enhancing therapeutic efficacy (Figure 6b,c). On top of that, oil red O staining of the aortic root revealed that the plaque area in the HFD group and the SMC‐HA group was ≈51.08% and 51.06%, respectively. After additional treatment with US irradiation in mice of the SMC‐HA group, the plaque area was significantly reduced to ≈17.2% (Figure 6d,e). To assess plaque stability, Masson staining was performed on the sections of aortic roots, revealing that collagen content in the HFD and US group was ≈24.05% and 23.97%, respectively.

In contrast, collagen content increased significantly to ≈43.12% in the SMC‐HA + US group, indicating that SMC‐HA‐mediated SCT promoted plaque stability (Figure 6d,f). Overall, these results demonstrate that SMC‐HA‐mediated SCT significantly alleviates AS progression in both mice models induced by partial carotid artery ligation combined with HFD in *ApoE^−/−^* mice and by long‐term HFD feeding alone.

SMC‐HA exhibits markedly enhanced sonocatalytic properties relative to conventional sonosensitizers. For example, 5‐aminolevulinic acid, a precursor of protoporphyrin IX, requires metabolic conversion in vivo that mandates high‐dose administration (60 mg kg^−1^ by tail vein injection).^[^ ^16^ ^]^ Besides, strict light avoidance for 24 h is required to prevent skin damage after injection. In contrast, SMC‐HA formulation achieves good therapeutic effects at a lower dose (10 mg kg^−1^) without photosensitivity risks. Notably, while Cao et al. reported TiO_2_ cooperating with CuS‐mediated phototherapy to inhibit early plaque development, their study lacked evaluation against advanced lesions.^[^ ^27^ ^]^ Our engineered SMC‐HA addresses this critical gap by demonstrating dual therapeutic benefits in two *ApoE^−/−^* mice models, not only inhibiting plaque progression but also promoting regression of established plaques. This breakthrough not only validates the potential of SMC‐HA in plaque prevention and treatment but also provides novel insights for precise plaque management.

### 2.8 In Vivo Distribution and Biosafety Assessment of SMC‐HA

**Figure 7:**
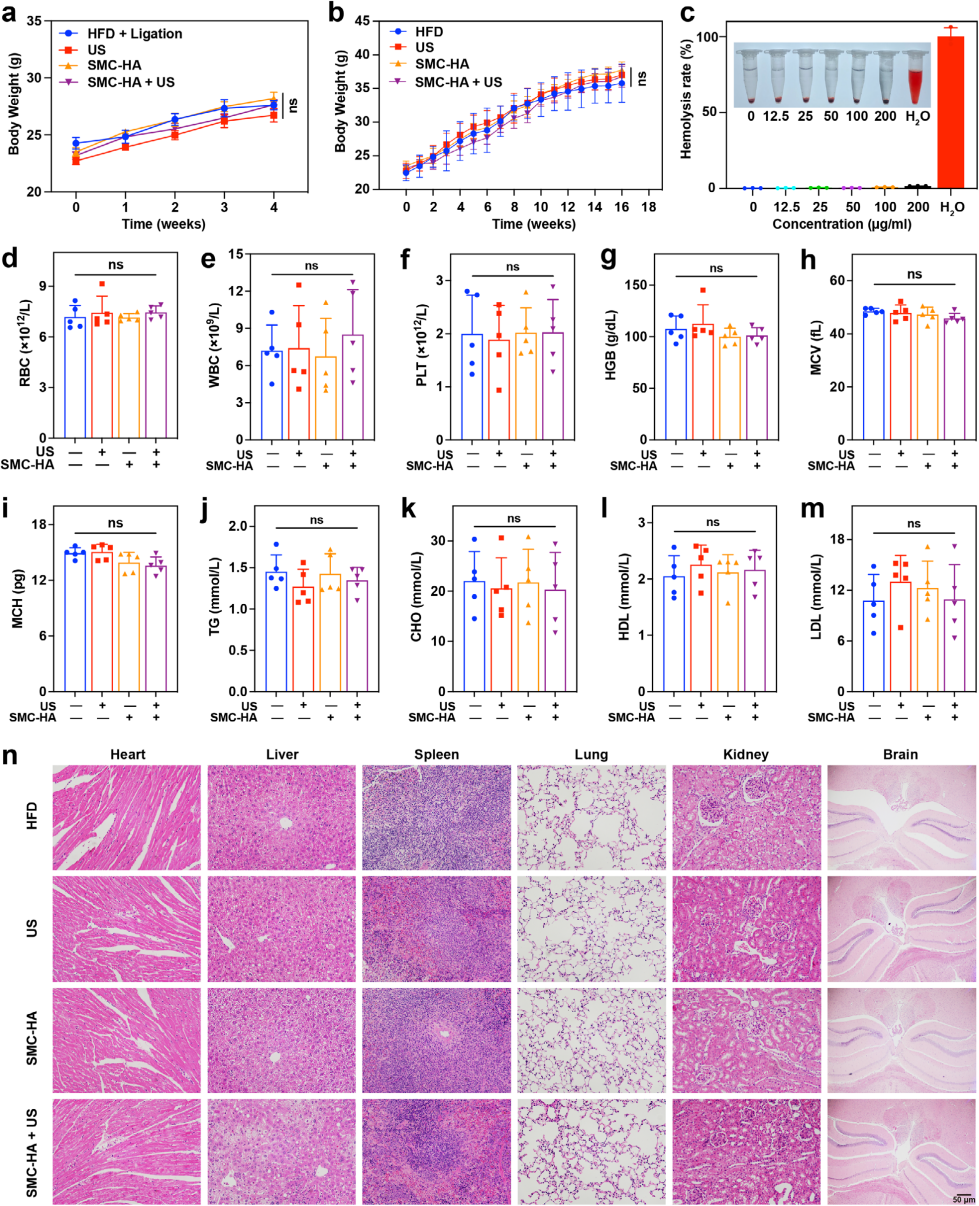
Biosafety assessment of SMC‐HA‐mediated SCT in two *ApoE^−/−^* mouse models of AS. a) Body weights of *ApoE* ^−/−^ mice with partial left carotid artery ligation and 4‐week high‐fat diet feeding (*n* = 5). b) Body weights of *ApoE* ^−/−^ mice with 14‐week high‐fat diet feeding (*n*  = 5). c) Hemolysis test of SMC‐HA in different concentrations (*n* = 3). d–i) The examination of blood routine parameters including d) RBC, e) WBC, f) PLT, g) HGB, h) MCV, i) MCH in *ApoE* ^−/−^ mice. j–m) The examination of serum lipid parameters including j) TG, k) CHO, l) HDL, m) LDL in *ApoE* ^−/−^ mice (*n* = 5). n) H&E staining images of the major organs (heart, liver, spleen, lung, kidney, and brain) in *ApoE* ^−/−^ mice after different treatments. Scale bar: 50 µm. ns = *p* > 0.05. Data are presented as mean values ± SD. ns: *p* > 0.05.

The safety of nanomaterials is a critical factor in their progression from preclinical to clinical trials. To evaluate the potential side effects of SMC‐HA in *ApoE^−/−^* mice, we monitored body weight fluctuations throughout the treatment duration. In both *ApoE^−/−^* mice models induced by partial carotid artery ligation combined with an HFD and those fed only HFD for 14 weeks, there were no notable variations in body weight between the treatment and control groups (**Figure** **7**a,b), suggesting that the overall health was unaffected. Next, we incubated fresh mouse blood with various concentrations of SMC‐HA, with PBS serving as a blank control. The results showed that concentrations of SMC‐HA up to 200 µg ml^−1^ did not cause hemolysis (Figure 7c). Post‐treatment blood samples were collected to assess potential blood toxicity (*n* = 5 mice per group). As shown in Figure 7d–i, no substantial differences were found in red blood cells (RBC), white blood cells (WBC), platelets (PLT), hemoglobin (HGB), mean corpuscular volume (MCV), or mean corpuscular hemoglobin (MCH). Likewise, no significant differences were observed in triglycerides (TG), cholesterol (CHO), low‐density lipoprotein (LDL), and high‐density lipoprotein (HDL), indicating that the anti‐atherosclerotic effects of SMC‐HA‐mediated SCT were not attributed to alterations in lipid profiles (Figure 7j–m). Additionally, a PerkinElmer IVIS Spectrum was used to track the SMC‐HA distribution in mouse organs. As shown in Figures S35a,b (Supporting Information), SMC‐HA predominantly accumulated in the liver and kidneys, with minimal expression in the heart, suggesting that it was primarily cleared via the liver and kidneys. Consistent with fluorescence imaging results, ICP‐MS data revealed that the majority of manganese was concentrated in the liver, followed by kidneys, and minimal cardiac deposition (Figure S35c, Supporting Information). Lastly, we conducted hematoxylin and eosin (H&E) staining on the major organs across all groups. No obvious tissue abnormalities were detected in the heart, liver, spleen, lungs, kidneys, and brain (Figure 7n). These findings confirmed that SMC‐HA‐mediated SCT does not induce local or systemic toxicity, supporting its potential as a safe and effective therapeutic approach for AS. The translational potential of SMC‐HA requires consideration of interspecies discrepancies, necessitating subsequent validation in other animal models and large‐scale preclinical studies. Apart from this, the US parameter optimization (intensity, frequency, and duty cycle) and regulatory pathways are also the key clinical translation considerations. In future investigation, priority should be given to long‐term therapeutic efficacy and safety profiles, including atherosclerotic plaque recurrence rates and chronic toxicity studies.

## 3 Conclusion

In conclusion, we successfully developed a microenvironment‐regulatable single‐atom sonozyme system, named SMC‐HA, a sonosensitizer with enzyme‐like activities, which exhibited a synergistic anti‐atherosclerotic effect under US irradiation. Our findings highlight several key advantages of SMC‐HA‐mediated SCT. First, by selectively targeting and binding to CD44 receptors, SMC‐HA preferentially accumulated in M1 macrophages within atherosclerotic plaques. This targeted delivery significantly enhanced therapeutic specificity while minimizing off‐target effects. Second, its CAT‐like and POD‐like activities contributed to microenvironmental modulation by reducing excessive H_2_O_2_ levels and alleviating hypoxia within the plaque microenvironment. Simultaneously, it promoted ·OH generation, thereby amplifying ROS‐mediated apoptosis in M1 macrophages, leading to substantial plaque regression. Furthermore, SMC‐HA‐mediated SCT demonstrated robust therapeutic efficacy in two independent animal models of atherosclerosis, effectively inhibiting disease progression and promoting plaque stabilization. Its dual role in both preventing and treating atherosclerosis underscores its translational potential and reproducibility across different pathological conditions. Notably, no systemic toxicity or adverse effects were observed in any treated mice, confirming its excellent biocompatibility and biosafety. Overall, this study establishes SMC‐HA‐mediated SCT as a promising, non‐invasive therapeutic strategy for atherosclerosis. By integrating targeted macrophage depletion with microenvironmental regulation, this approach effectively mitigates plaque burden while improving vascular health. Given its superior therapeutic performance and high safety profile, SMC‐HA holds significant potential for future clinical translation as an advanced nanoplatform for the treatment of atherosclerotic cardiovascular diseases.

## 4 Experimental Section

### Synthesis of ZIF‐8 and CNF

1.071 g of Zn (NO_3_)_2_·6H_2_O was mixed with 40 mL of methanol to form solution A, while 2.357 g of 2‐methylimidazole was combined with another 40 mL of methanol to prepare solution B. Next, solution A was added to solution B while stirring, and the mixture was continuously stirred at ambient temperature overnight. The resulting product was rinsed with methanol and heated at 80 °C for 12 h to obtain ZIF‐8 nanocrystals. Finally, 200 mg of ZIF‐8 nanocrystals were placed under a nitrogen environment at a flow rate of 100 sccm, heated to 950 °C, and calcined for 2.5 h to yield CNF.

### Synthesis of SMC

To prepare SMC, 50 mg MnCl_2_ was ultrasonically dispersed in 40 mL CNF solution, followed by methanol washing and drying. The obtained powder was then calcined at 750 °C, cooled to room temperature, and collected as the SMC powder for further use. The manganese loading percentage was quantitatively determined to be 1.7 wt.% through inductively coupled plasma optical emission spectroscopy (ICP‐OES).

### Preparation of SMC‐HA, SMC‐Cy5.5, and SMC‐HA‐Cy5.5

First, 10 mg of SMC powder was dispersed in 10 mL of chloroform through 30 min of sonication. Next, 30 mg of DSPE‐PEG and HA was added to the mixture, followed by an additional 5 min of ultrasonication. After further evaporating under nitrogen flow, the sample was dried in a vacuum oven. After that, the mixture was ultrasonicated for 5 min with ultrapure water. The final dispersion was subjected to centrifugation and washing to obtain the SMC‐HA product. To obtain Cy5.5‐labeled SMC and SMC‐HA (shown as SMC‐Cy5.5 and SMC‐HA‐Cy5.5), DSPE‐PEG was substituted with DSPE‐PEG‐Cy5.5.

### Sonocatalytic Activity Assay

A stock solution of CNF and SMC at 1 mg mL^−1^ was adjusted with ultrapure water to 100 µg mL^−1^. Baseline absorption was initially recorded using a UV–vis–NIR instrument. Subsequently, 20 µg mL^−1^ ABDA was added, and the mixture was subjected to US irradiation (1.0 W cm^−2^, 1.0 MHz, 50% duty cycle) in the dark. The changes in ABDA absorption intensity were recorded at 2‐min intervals for a total of 10 min. Additionally, TEMP served as a reagent to capture ^1^O_2_ for ESR spectroscopy. A 5 µL aliquot of TEMP (100 mm) was added to 100 µL of either CNF or SMC (50 µg mL^−1^) solution, followed by US irradiation (1.0 W cm^−2^, 1.0 MHz, 50% duty cycle) for 2 min. Finally, 2 µL of the mixture was injected into a quartz capillary tube, and ESR spectroscopy was immediately performed to measure the ^1^O_2_ signal. To evaluate the ·OH‐generating ability of US‐activated SMC, MB was utilized as a probe. MB (1.0 mg mL^−1^, 40 µL) and SMC (100 µg mL^−1^) were combined with deionized water. Next, the mixture was exposed to US irradiation for 15 min, and 665 nm peak was detected every 3 min using UV–vis–NIR spectroscopy.

### Enzyme‐Like Activity Assay

To evaluate the CAT‐like activity of nanomaterials, SMC, CNF, and MnO_2_ were each added to 5 mL of PBS buffer to achieve final concentrations of 25 µg mL^−1^. Subsequently, 1 mm H_2_O_2_ was added, and the dissolved oxygen concentration of the reaction solution was promptly recorded by a portable dissolved oxygen meter. Data were recorded every 30 s over a continuous 15‐min period. Buffer solutions with concentrations of 0.1, 0.25, 0.5, and 1 mm H_2_O_2_ were prepared to assess the H_2_O_2_‐concentration‐dependent effect. To assess the POD‐like ability of SMC‐HA, MnO_2_, CNF, or SMC (50 µg mL^−1^) were prepared in an acetate/sodium acetate buffer. TMB and H_2_O_2_ were subsequently incorporated to reach 100 µm for each reagent. The absorbance at 652 nm was recorded using a UV–vis–NIR instrument, with readings taken every 10 s over a 200‐s period to track the reaction kinetics.

### Cell Culture

RAW 264.7 macrophages were cultured in DMEM medium (10% fetal bovine serum and 1% penicillin/streptomycin). L929 cells were cultured in MEM medium (10% fetal bovine serum and 1% penicillin/streptomycin). All cells were grown in a 37 °C incubator (5% CO_2_, 20% O_2_, 75% N_2_) in a humidified environment. To activate the cells into proinflammatory M1 macrophages, RAW264.7 macrophages were incubated with 1 µg mL^−1^ LPS for 12 h.

### The Isolation and Differentiation of Mouse BMDMs

Bone marrow‐derived macrophages (BMDMs) were isolated and differentiated following the protocol established by Edgar et al.^[^ ^33^ ^]^ Briefly, L929 cell‐conditioned medium was prepared by culturing cells in MEM medium for 7–8 days until confluence, followed by medium collection and storage. BMDMs were then aseptically flushed from murine femurs and tibias and differentiated for 7 days in specialized medium comprising 70% DMEM, 10% fetal bovine serum, 1% antibiotics, and 20% L929 cell‐conditioned medium. For phenotypic polarization, differentiated macrophages were treated with either 100 ng mL^−1^ LPS (M1 polarization) or 10 ng mL^−1^ recombinant murine IL‐4 (M2 polarization).

### Cellular Uptake In Vitro

RAW264.7 macrophages in the logarithmic phase were seeded in culture dishes (10 × 10^4^ cells mL^−1^) and divided into three groups: SMC‐HA, LPS + SMC, and LPS + SMC‐HA. In the LPS + SMC and LPS + SMC‐HA groups, 1 µg mL^−1^ of LPS was added and incubated for 12 h, while the SMC‐HA group was treated with PBS. Next, the spent culture medium was removed, and the cells were rinsed with PBS. Cy5.5‐labeled SMC or SMC‐HA was added at the time points of 0, 2, 4, 8, 12, and 24 h. Finally, all cells were stained with Hoechst 33342 for 10 min and observed under the CLSM.

To quantitatively assess nanoparticle uptake across macrophage phenotypes, Cy5.5‐conjugated SMC and SMC‐HA nanoparticles were incubated for 8 hours with polarized BMDMs (M0/M1/M2), followed by nuclear staining with Hoechst 33342. Finally, Cy5.5‐labeled nanoparticles in BMDMs were detected by flow cytometry.

### Cytotoxicity Evaluation

Cell viability after incubation with SMC‐HA was evaluated by the CCK‐8 assay. RAW264.7 macrophages were seeded into 96‐well plates and treated with LPS to induce proinflammatory status. The old medium was then replaced with different concentrations of SMC‐HA (0, 6.25, 12.5, 25, 50 µg mL^−1^) for 8 h. Next, cells were incubated for an additional 30 min after adding 10 µL of CCK‐8 reagent, and the absorbance at 450 nm was recorded. In addition, the CCK‐8 method was used to assess cell viability in different experimental groups, including those with or without US irradiation (0.5 W cm^−2^), and with different US intensities (0.5, 0.8, 1.0 W cm^−2^, 1.0 MHz, 50% duty cycle). US exposure lasted for 10 min, after which the cells were further incubated for 24 h before measuring viability. For BMDM cells, the post‐differentiation treatment procedures were identical to those for RAW264.7 cells.

### Live/Dead Cell Staining Assay

To further validate the efficacy of SMC‐HA‐mediated SCT in cells, M1 macrophages were categorized into four groups: 1) Control; 2) US; 3) SMC‐HA; 4) SMC‐HA + US. After undergoing various treatments, macrophages were incubated with PI and Calcein‐AM for 30 min. Following PBS washing, the macrophages were observed using fluorescence microscopy.

### Apoptosis Analysis

RAW264.7 macrophages after LPS stimulation were categorized into four groups: 1) Control; 2) US; 3) SMC‐HA; 4) SMC‐HA + US. Each group received either PBS or SMC‐HA (25 µg mL^−1^). The US and SMC‐HA + US groups were subjected to 0.5 W cm^−2^ US irradiation for 10 min, after which the cells were cultured in 37 °C incubator (5% CO_2_, 20% O_2_, 75% N_2_) for 24 h. Following the treatments, inflammatory macrophages were collected and resuspended in PBS. Next, inflammatory macrophages incubated with Annexin‐V and PI for 20 min in the dark, and apoptosis analysis was measured using flow cytometry.

To elucidate the ROS‐mediated apoptotic pathway, cells were divided into several groups. The ROS scavenger was added 30 min prior to SMC‐HA treatment at a final dilution ratio of 1:500, with subsequent US irradiation and apoptosis assay procedures performed as previously described.

### Intracellular ROS Detection

Inverted fluorescence microscopy and flow cytometry analysis were used to assess ROS production in LPS‐activated RAW 264.7 macrophages. Cells were categorized into four groups: 1) Control; 2) US; 3) SMC‐HA; 4) SMC‐HA + US. After treatment, the culture medium was replaced with serum‐free medium containing DCFH‐DA (10 µm) and incubated in the dark for 30 min. After being stained with Hoechst 33342, fluorescence microscopy was used to observe and capture images. For flow cytometry analysis, the macrophages were suspended in 200 µL of PBS and subsequently placed in flow cytometry tubes for detection.

### Intracellular O_2_ Detection Induced by SMC‐HA

[Ru(dpp)_3_]Cl_2_ is a well‐known O_2_‐sensitive probe, which exhibits fluorescence quenching upon interaction with O_2_.^[^ ^34^ ^]^ First, M1 macrophages were seeded in confocal dishes and grown under hypoxic conditions (1% O_2_, 5% CO_2_, 94% N_2_) for 24 h. Following this, the cells were incubated with [Ru(dpp)_3_]Cl_2_ for 4 h, and then with SMC‐HA (25 µg mL^−1^) for an additional 8 h. Lastly, cells were stained with Hoechst 33342 for nuclear visualization, and intracellular fluorescent signals were photographed by CLSM.

The oxygen‐generating capability of SMC‐HA was further validated in BMDMs, which were allocated into five experimental conditions. Among them, hypoxic induction was achieved using a hypoxic culture chamber, while control groups were maintained under standard culture conditions. [Ru(dpp)_3_]Cl_2_ and SMC‐HA treatments were performed as previously described. Finally, cells were stained with Hoechst 33342 and observed under an inverted fluorescence microscope.

### Western Blot Analysis

Cells from different groups were put on ice with RIPA for cell lysis. The protein concentration was then determined by the BCA Protein Assay kit. Using 8% SDS‐PAGE, the total proteins were separated and then blotted onto a 0.22 µm PVDF membrane. After blocking the membrane with a protein‐free rapid blocking solution at room temperature for 30 min, it was incubated with primary antibodies overnight at 4 °C: rabbit anti‐Bcl‐2 (1:1000), rabbit anti‐Bax (1:1000), mouse anti‐HIF‐1α (1:2000), and rabbit anti‐β‐actin (1:1000). The membrane was incubated with horseradish peroxidase (HRP)‐conjugated secondary antibodies (1:3000) after rinsing with Tris‐Buffered Saline with Tween 20 (TBST) for three times. Using the enhanced chemiluminescence (ECL) system, protein bands were visualized, and the intensities of the bands were quantified with ImageJ software.

### Quantitative RT‐PCR

RNA was extracted from cells using Trizol reagent, and its concentration was quantified with the Nanodrop 2000 system. Subsequently, the total RNA was reverse transcribed into cDNA using a reverse transcription kit. The cDNA was then amplified in the LightCycler 480 Real‐Time PCR System using SYBR qPCR Master Mix. The primer sequences used for real‐time PCR are listed in Table S1 (Supporting Information). The results were analyzed using the LightCycler 480 analysis software, with data presented as Ct values. Using the computational formula of 2^−ΔΔCt^, gene expression levels were calculated and normalized to GAPDH.

### Animal Models

The Ethics Committee of Sun Yat‐sen Memorial Hospital, Sun Yat‐sen University, approved all animal experiments (ethical approval number AP20240160). *ApoE^−/−^* mice (6‐8 weeks old, male) were gained from GemPharmatech Co., Ltd. The *ApoE^−/−^* mice were used to establish two atherosclerotic plaque models: 1) carotid atherosclerotic plaques induced by partial carotid artery ligation combined with an HFD for 4 weeks, and 2) aortic atherosclerotic plaques developed through HFD feeding alone for 14 weeks. Partial ligation of the left carotid artery was carried out to form plaque in 28 days. Briefly, mice were given a HFD for 14 days. Subsequently, the left carotid artery was exposed, and three out of its four caudal branches (including internal carotid artery, external carotid artery, and occipital artery) were tied off with 8−0 silk sutures. Finally, HFD feeding continued for another 14 days, and the mice were euthanized on day 28. The LCCA was harvested, showing significant plaque formation. For the “aortic atherosclerotic plaque” model, *ApoE^−/−^* mice were fed a HFD for 14 weeks. On week 14, the entire aorta from mice was harvested, displaying multiple white plaques of varying sizes within the aortic vessel. These results confirm the successful establishment of the carotid atherosclerotic plaque and aortic atherosclerotic plaque models, which can be used for subsequent targeting and therapeutic experiments.

### Micro‐Ultrasound Imaging

Ultrasound imaging parameters of the LCCA were assessed using the Vevo 2100 imaging system. Mice were anesthetized and securely positioned on the operating platform. The fur on their necks was carefully shaved, and a generous amount of warm ultrasound coupling gel was applied to ensure optimal image quality. Initially, the imaging mode was set to B‐mode, capturing the long‐axis and short‐axis view of LCCA. Next, the M‐mode was used to observe the contraction and relaxation of the vessel. The software analysis allowed for measuring the Ds and Dd values. Finally, the probe was maintained at an angle of ≈60° to the mouse neck, and the angle correction line was aligned with the direction of blood flow. The system was switched to PW mode to measure blood flow velocity. The value of RI was determined using the formula: [PSV – EDV] / PSV, after recording the peak systolic velocity (PSV) and end‐diastolic velocity (EDV).

### In Vivo Localization of SMC‐HA

For the carotid atherosclerotic plaque model, 14 days after partial carotid artery ligation surgery, mice were intravenously injected with SMC‐HA‐Cy5.5. Fluorescence expression in the neck region was monitored at different time points (0, 4, 8, 12, 24, 48, and 72 h) using the PerkinElmer in vivo imaging system. The remaining mice were randomly assigned to receive PBS, SMC‐Cy5.5 (10 mg kg^−1^), or SMC‐HA‐Cy5.5 (10 mg kg^−1^) via tail vein injection. The LCCA, aortic arch, and major organs were carefully excised after 24 h. Fluorescence intensity in the carotid artery and major organs was quantified using Living Image version 4.4 software. To further assess the co‐localization of SMC‐HA‐Cy5.5 with M1 macrophages in the carotid plaques. After intravenous injection, the LCCA was dissected for frozen section preparation. Immunofluorescence staining was performed on arterial tissue, with the M1 macrophage marker CD86 labeled in green fluorescence, nuclei in blue fluorescence, and red fluorescence representing the SMC‐HA. For the aortic atherosclerotic plaque model, PBS or SMC‐Cy5.5 and SMC‐HA‐Cy5.5 were intravenously injected into mice fed an HFD for 14 weeks. 24 h later, the entire aorta was dissected, and imaged using the PerkinElmer in vivo imaging system for further analysis.

### In Vivo Therapeutic Effects

After carotid artery ligation, mice were randomly assigned to four groups: 1) Control; 2) US; 3) SMC‐HA; 4) SMC‐HA + US. On days 17, 21, and 25, mice were intravenously injected with SMC‐HA (10 mg kg^−1^) or PBS via tail vein injection. 24 h after the injection, the mice were anesthetized and placed in the supine position, accepting US irradiation (1.0 W cm^−2^, 1.0 MHz, 50% duty cycle). On day 28, the LCCA was then excised, fixed, sectioned, and stained. For the atherosclerotic aorta model, after 14 weeks of HFD feeding, mice were similarly divided into four groups. Over the following week, mice were intravenously injected with SMC‐HA (10 mg kg^−1^) or PBS via tail vein injection. 24 h after administration, targeted US irradiation (1.0 W cm^−2^, 1.0 MHz, 50% duty cycle) was applied sequentially to three distinct anatomical sites (thoracic, upper abdominal, and lower abdominal regions), with 10 min exposure per site. This treatment was repeated seven times, with the “SMC‐HA injection” and “US irradiation” procedure performed every 24 h. After all treatments, the mice were euthanized, and the aortas were excised for gross Oil Red O staining. The aortic root was further processed for Oil Red O and Masson staining.

### Biosafety Analysis

Starting from the first day of feeding, the body weight of all mice was recorded weekly until euthanasia. For each experimental group, 5 mice were allocated. After finishing experiment, all mice were humanely euthanized for collection of whole blood and serum specimens. To assess intergroup variations in hematological parameters and lipid profiles across the four experimental groups, initial evaluation of data distribution normality and variance homogeneity, followed by one‐way analysis of variance (ANOVA). Finally, the major organs were excised for histological examination using H&E staining.

### Statistical Analysis

GraphPad Prism 8.0 software was used for data handling and statistical evaluation, with each experiment repeated at least three times. The data were expressed as mean ± SD. Student's t‐test was used to evaluate differences between two independent groups, and one‐way or two‐way ANOVA was applied for comparisons among multiple groups. Statistical significance is indicated as follows: ns: *p* > 0.05, *p < 0.05, **p < 0.01, ***p < 0.001, and ****p < 0.0001.

## Conflict of Interest

The authors declare no conflict of interest.

## Author Contributions

Q.C. and G.Y. contributed equally to this work. Y.P. and Y. Z. were responsible for the entire project and revised the draft. Q.C. and G.Y. collaboratively conducted the synthesis of materials and the subsequent cellular and animal experiments, and they prepared the initial manuscripts together. Z. L. and Z. C. assisted with part of the experiment. L.H., R.L., and T.H. helped process the raw data. M.Z., K.W., and C.H. critically revised and edited the English expressions in manuscript. S.Z., P.Z., and J.W. helped check the final version of this paper.

## Supporting information



Supporting Information

## Data Availability

The data that support the findings of this study are available from the corresponding author upon reasonable request.
