# Supplementary material for: A Hypoxia‐Responsive Single‐Atom Sonozyme for Targeted Sonocatalytic Therapy in Alleviating Atherosclerotic Plaque
Source: Adv Sci (Weinh). 2025 Sep 23;12(46):e05058. doi: 10.1002/advs.202505058 (PMC12697858; doi:10.1002/advs.202505058)
Supplement: Supplementary file 1 — Supporting Information [file ADVS-12-e05058-s001.docx]

**Supporting Information**

**A Hypoxia-Responsive Single-Atom Sonozyme for Targeted Sonocatalytic Therapy in Alleviating Atherosclerotic Plaque**

Qiaofei Chen^#^, Guotao Yuan^#^, Zhiwen Liu, Zhengyu Cao, Li He, Ruonan Li, Tongsheng Huang, Minglong Zheng, Kexin Wen, Canxia Huang, Shuang Zhu, Pingyu Zhang, Jingfeng Wang, Yuling Zhang* and Yue Pan*

**Q. Chen, G. Yuan, Z. Liu, Z. Cao, L. He, R. Li, T. Huang, M. Zheng, K. Wen, C. Huang, J. Wang, Y. Zhang, Y. Pan**

Department of Cardiology, Guangdong Provincial Key Laboratory of Malignant Tumor Epigenetics and Gene Regulation, Guangdong-Hong Kong Joint Laboratory for RNA Medicine, Medical Research Center, Sun Yat-sen Memorial Hospital, Sun Yat-sen University, Guangzhou 510120, China

**Z. Liu, S. Zhu**

School of Life Sciences and Biopharmaceutics, Guangdong Pharmaceutical University, Guangzhou, 510006, China

**G. Yuan, P. Zhang**

College of Chemistry and Environmental Engineering, Shenzhen University, Shenzhen, 518037, China

**G. Yuan**

Department of Otolaryngology, Longgang E.N.T. Hospital & Shenzhen Key Laboratory of E.N.T. Shenzhen 518116, China

***Correspondence author**: zhyul@mail.sysu.edu.cn, panyue@mail.sysu.edu.cn

^#^These authors contributed equally to this work.

**Supplementary Figures**


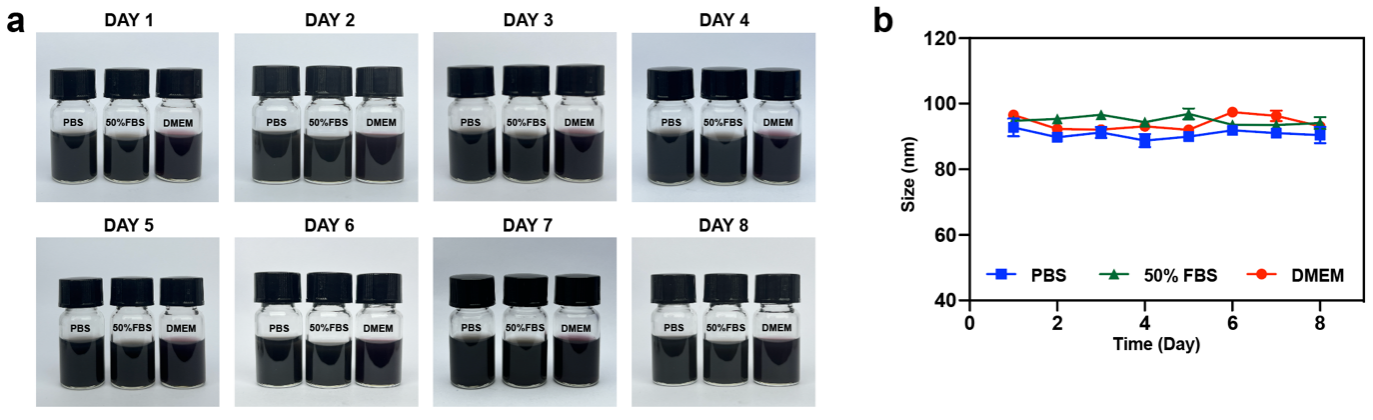


**Figure S1.** (a) Photo images of SMC in PBS, 50% FBS, and DMEM solution at different time points. (b) The hydrodynamic diameters of SMC in PBS, 50% FBS, and DMEM solution at different time points.

**
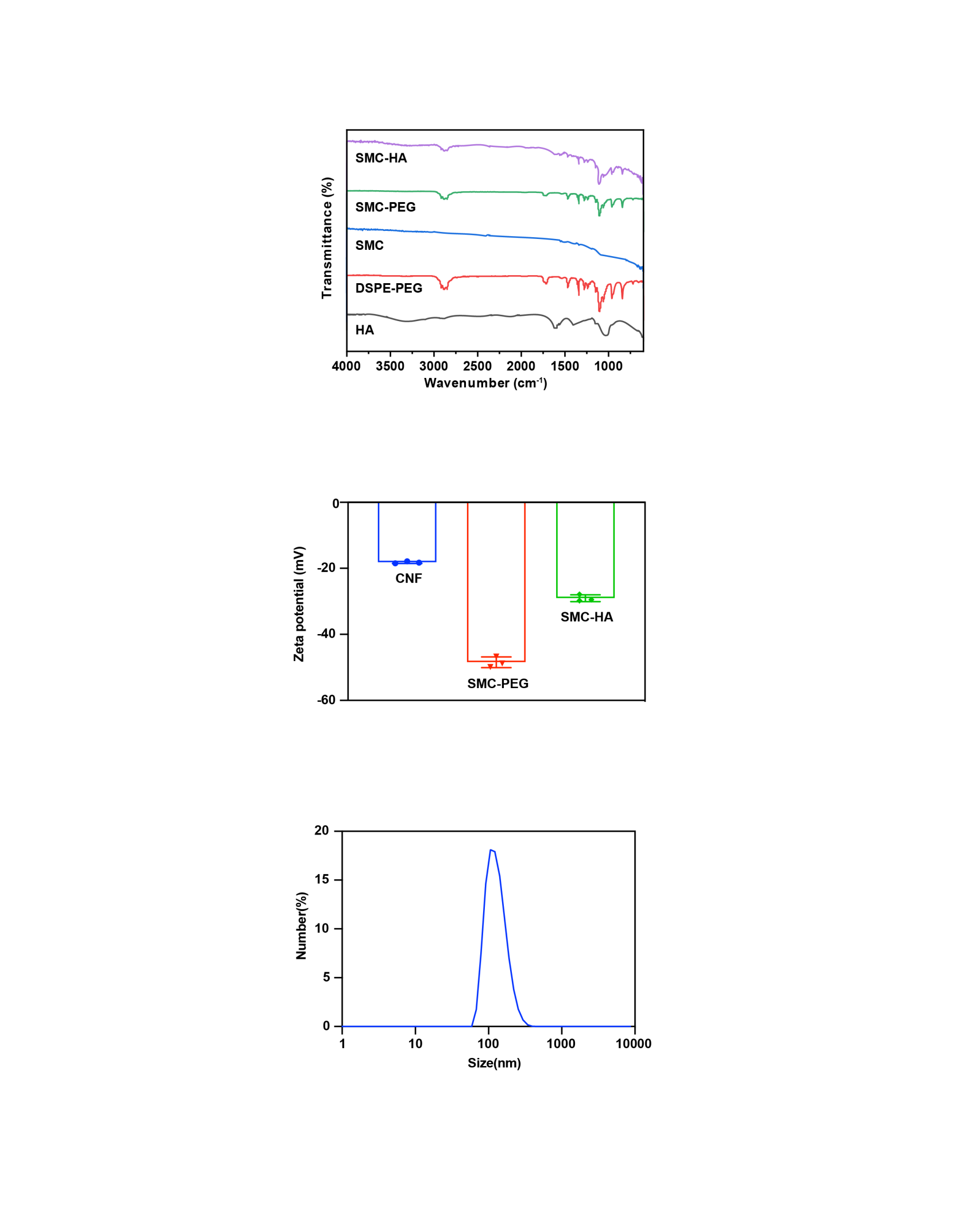
**

**Figure S2.** FT-IR of SMC-HA, SMC-PEG, SMC, DSPE-PEG and HA.

**
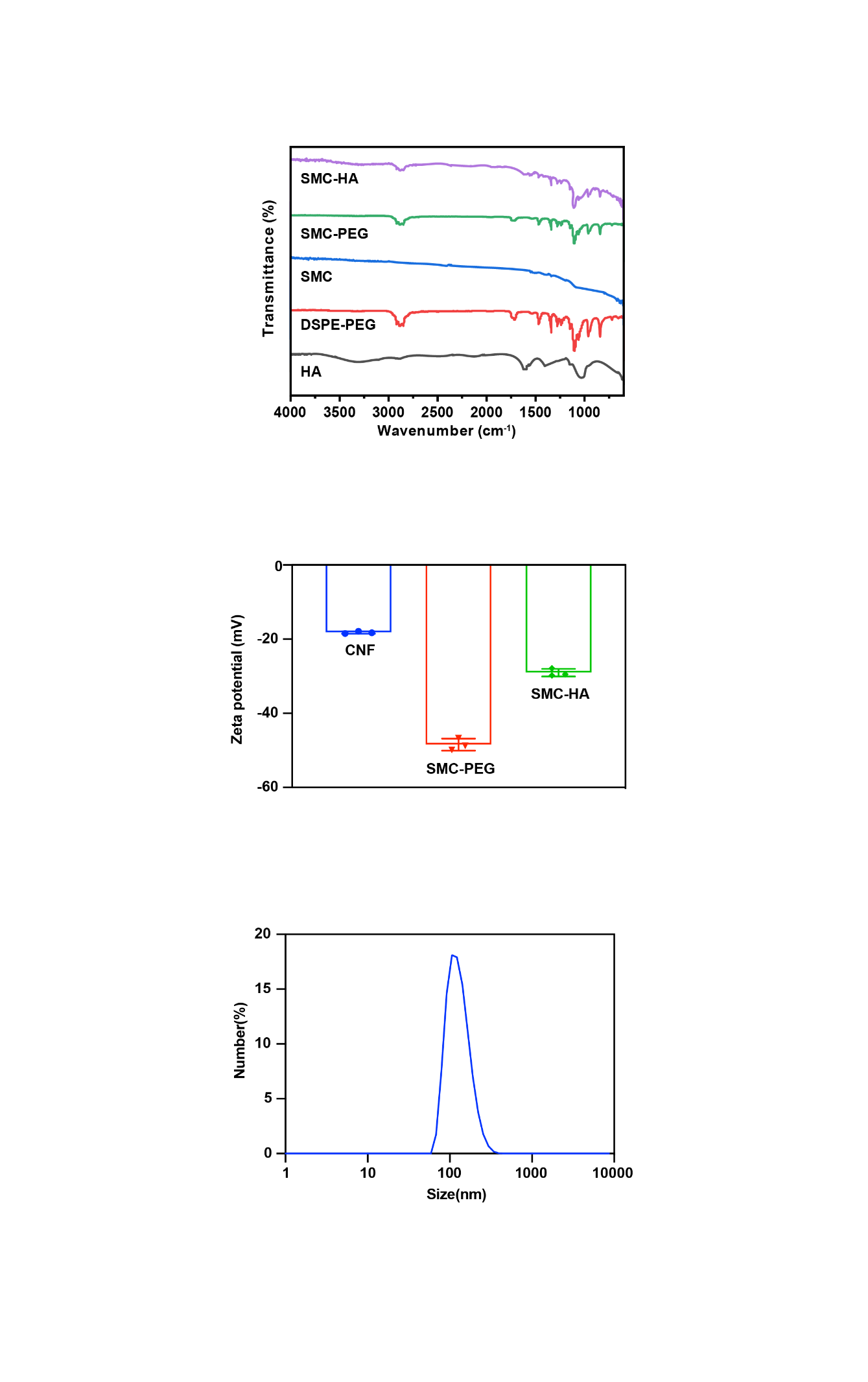
**

**Figure S3.** Zeta potential of CNF, SMC-PEG and SMC-HA (n = 3).

**
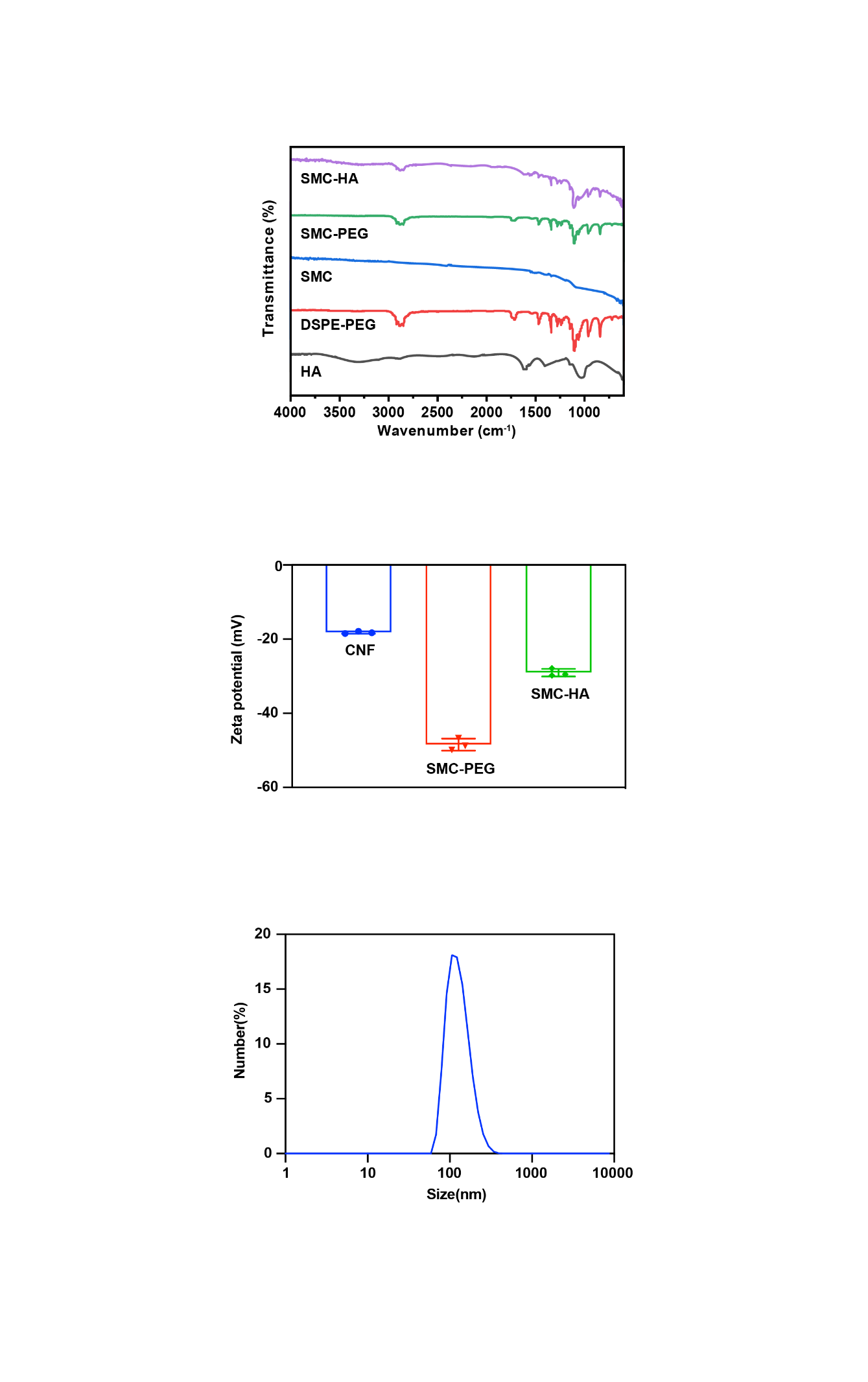
**

**Figure S4.** DLS of SMC-HA.

**
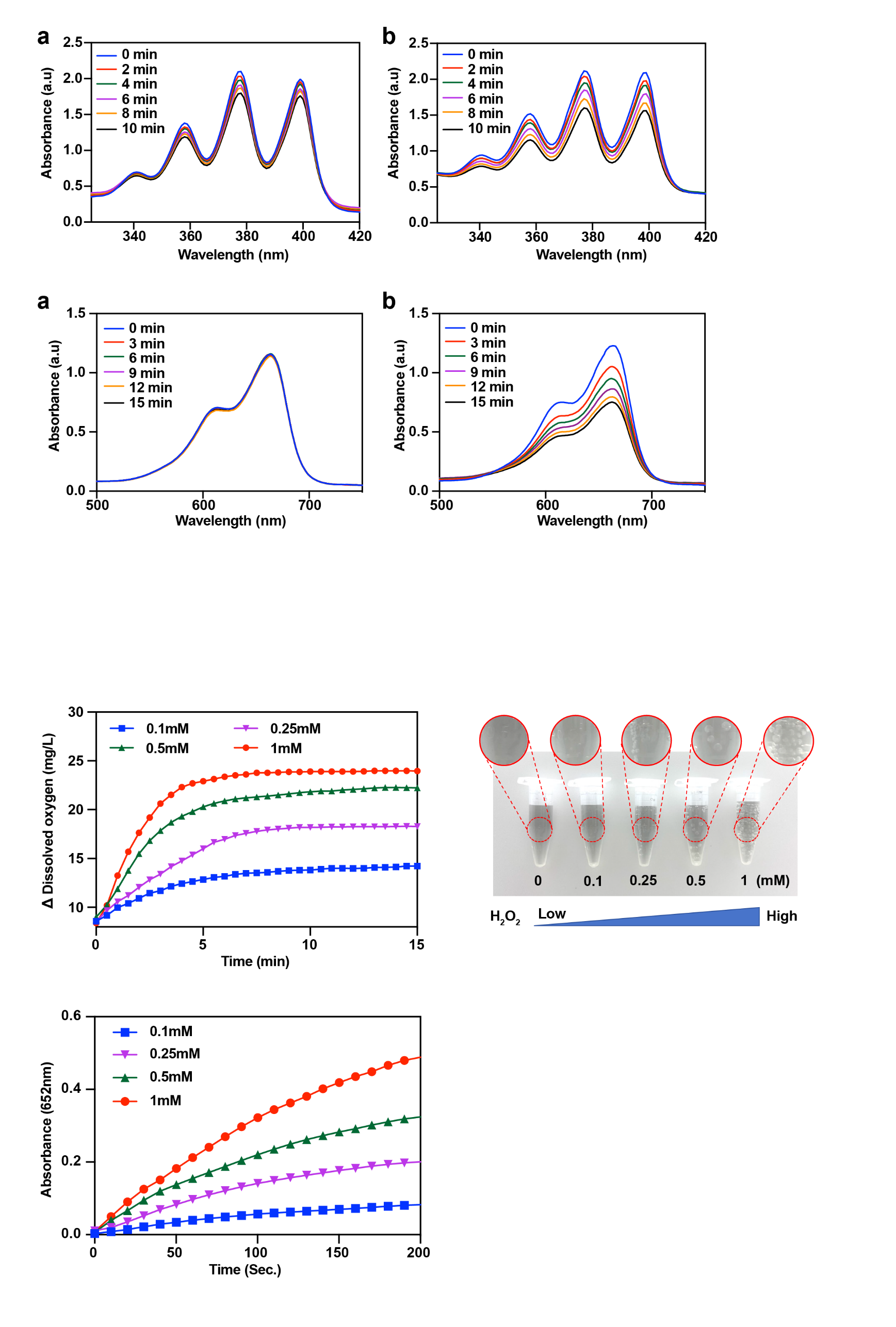
Figure S5.** The absorption of ABDA in (a) PBS + US and (b) CNF + US groups.

**
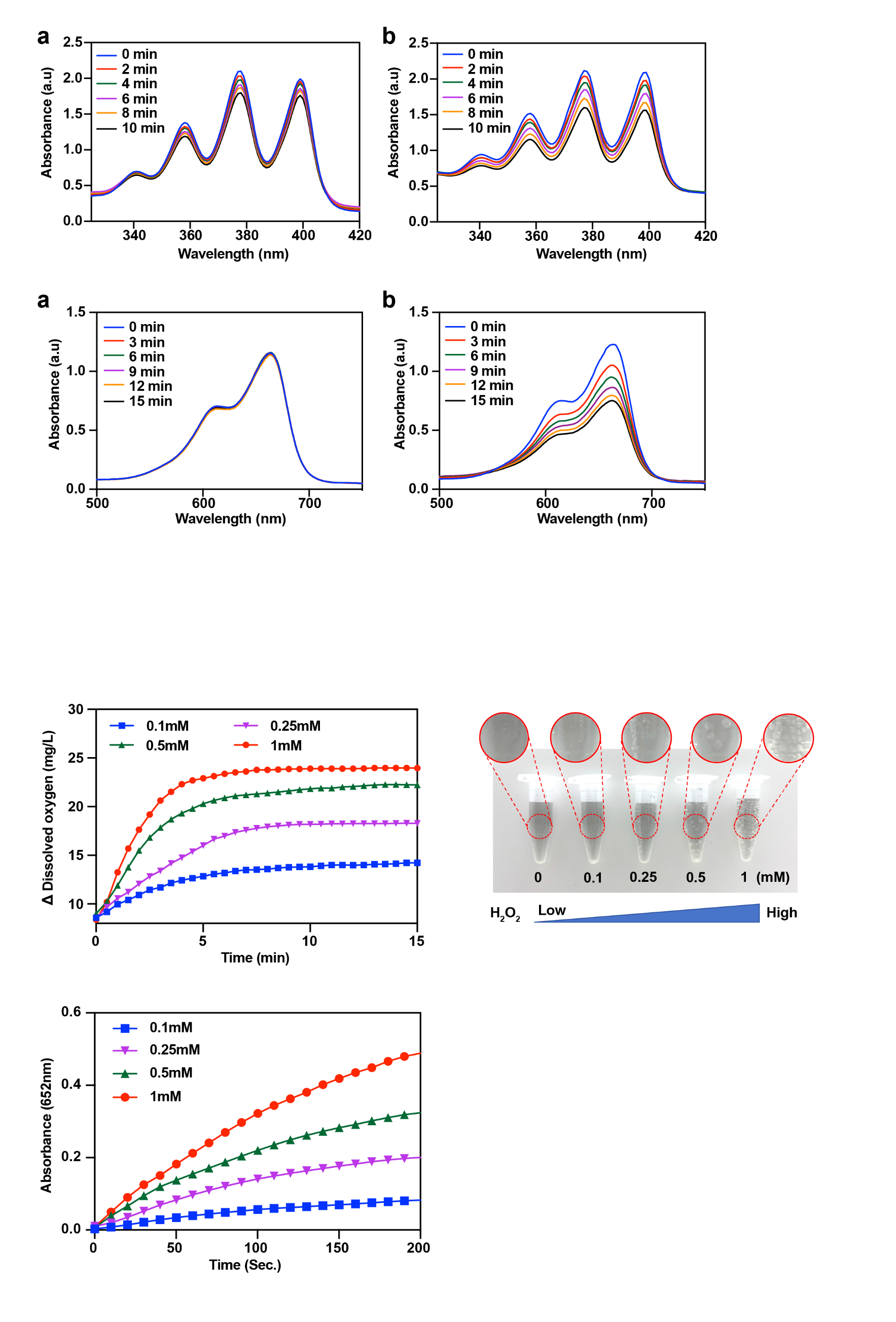
**

**Figure S6.** The absorption of MB in (a) PBS + US and (b) CNF + US groups.


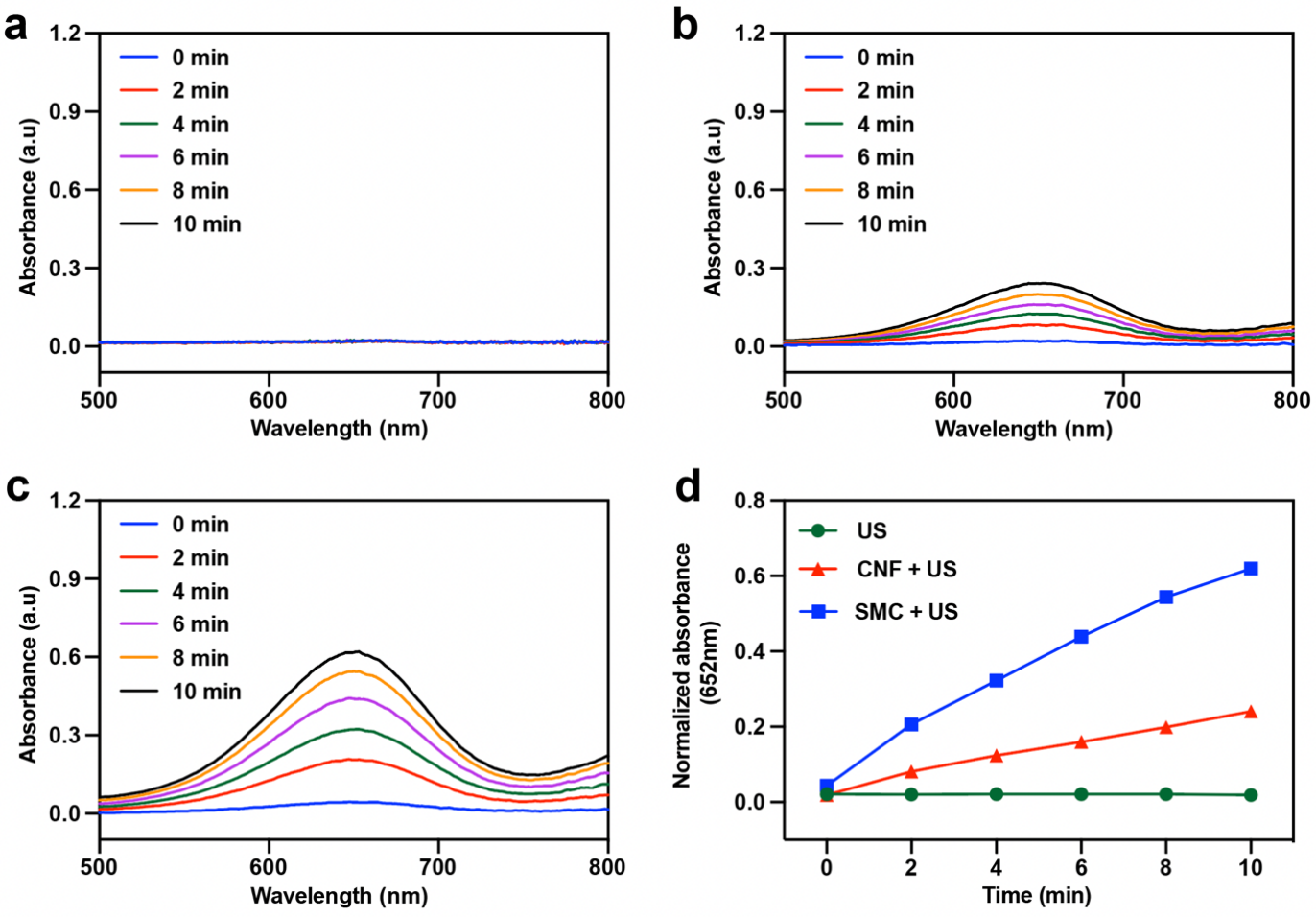


**Figure S7.** The UV-vis absorbance spectra of TMB in the (a) PBS + US group, (b) CNF + US group and (c) SMC + US group over time. (d) The normalized absorption at 652 nm following treatment with PBS, CNF, and SMC followed by US irradiation for 10 min.

**
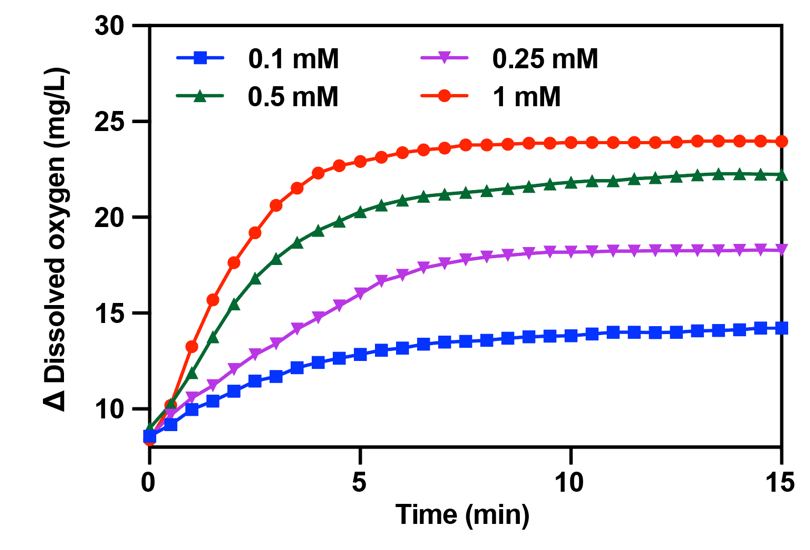
**

**Figure S8.** Comparison of O_2_ generation treated with SMC in the elevated concentrations of H_2_O_2_ solution.

**
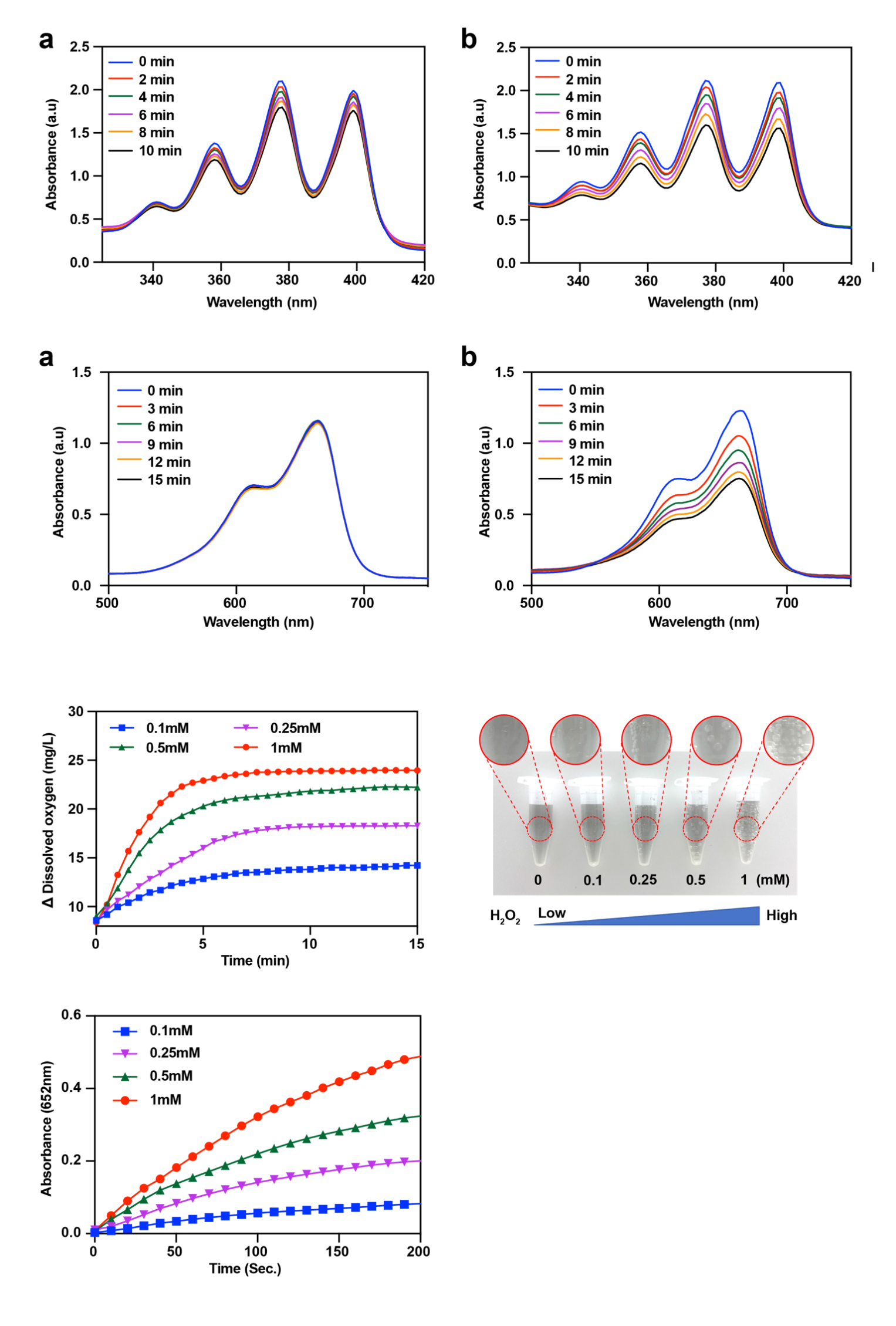
**

**Figure S9.** O_2_ bubbles on the inside of the tube wall treated with SMC in the elevated concentrations of H_2_O_2_ solution.

**
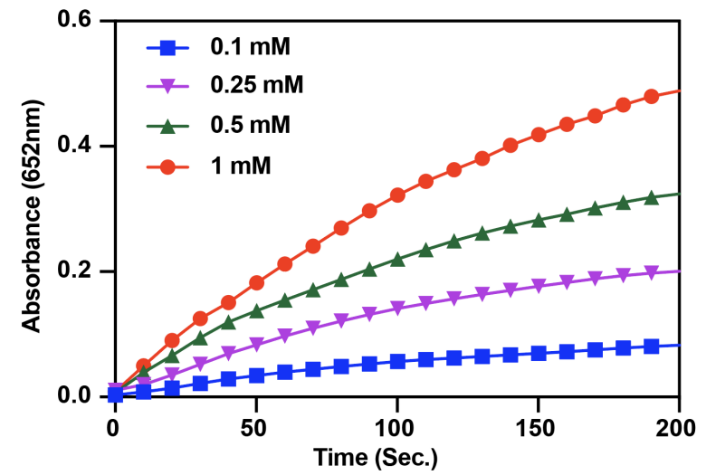
**

**Figure S10.** Comparison of ·OH generation treated with SMC in the elevated concentrations of H_2_O_2_ solution.

**
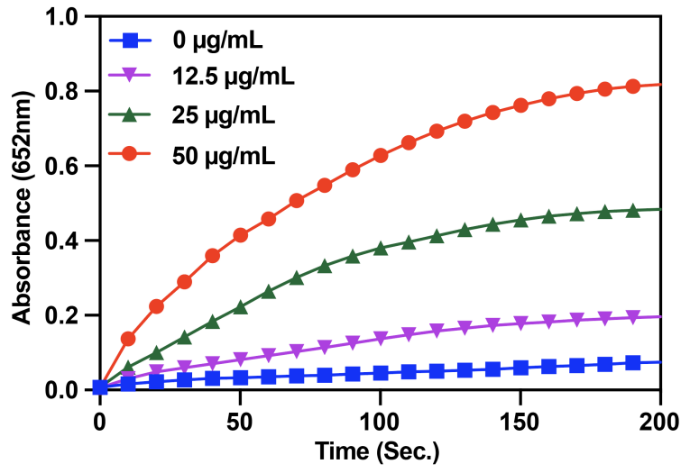
**

**Figure S11.** Comparison of ·OH generation treated with different SMC concentrations in the H_2_O_2_ solution.


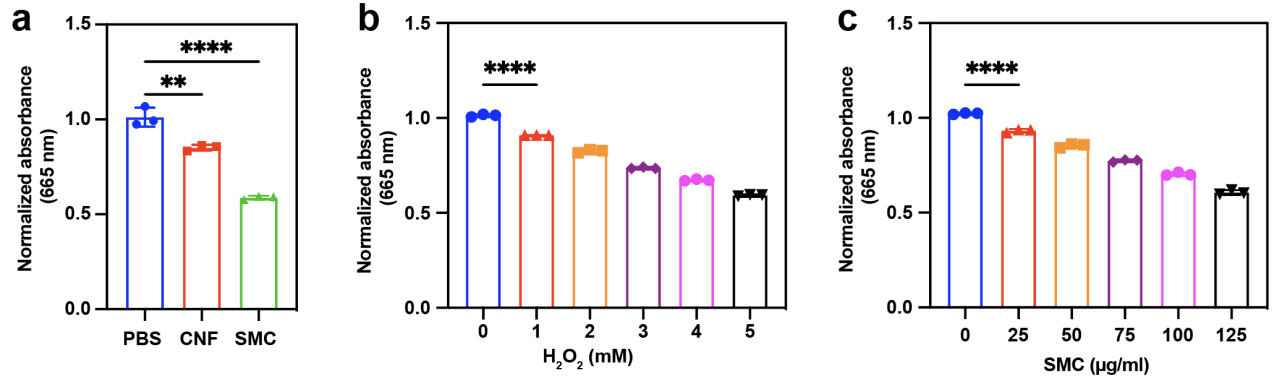


**Figure S12.** The absorption of MB at 665 nm evaluated under the following conditions: (a) PBS, CNF, SMC, (b) varying H_2_O_2_ concentrations, and (c) varying SMC concentrations (n = 3). Data are presented as mean values ± SD. ***p* < 0.01, *****p* < 0.0001.


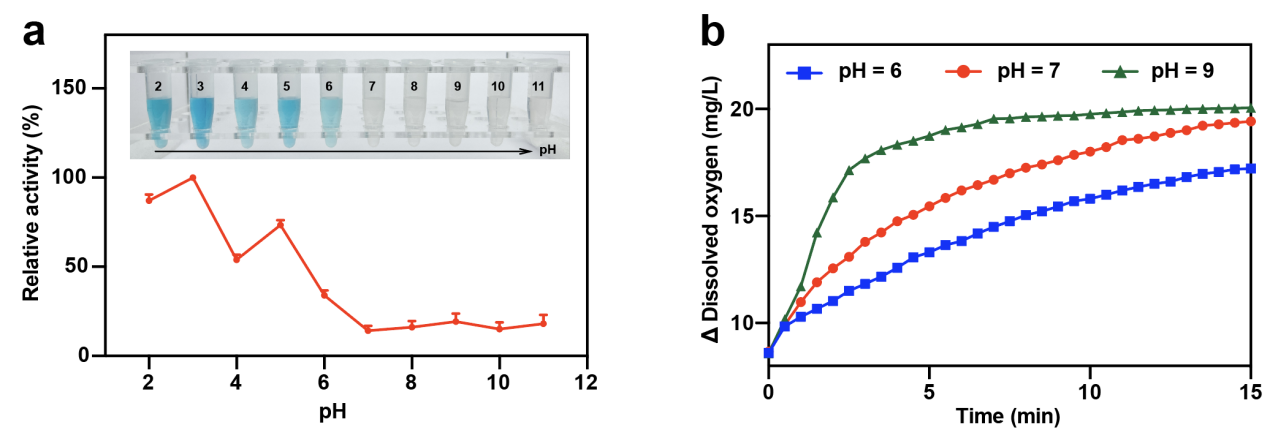


**Figure S13.** pH-dependent (a) POD-like and (b) CAT-like activities of SMC.


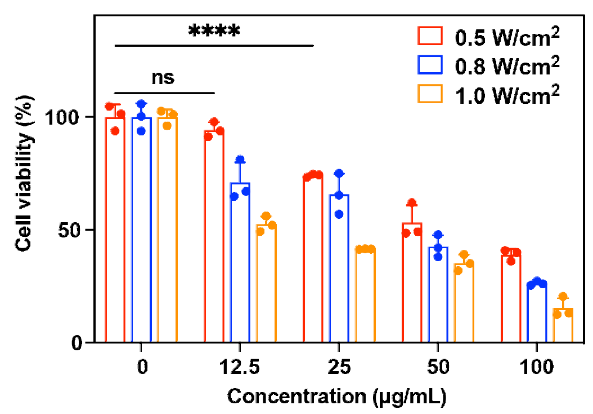


**Figure S14.** Cell viability of inflammatory macrophages incubated with SMC-HA at various concentrations and different US intensities (n = 3). Data are presented as mean values ± SD. ns: *p* > 0.05, *****p* < 0.0001.


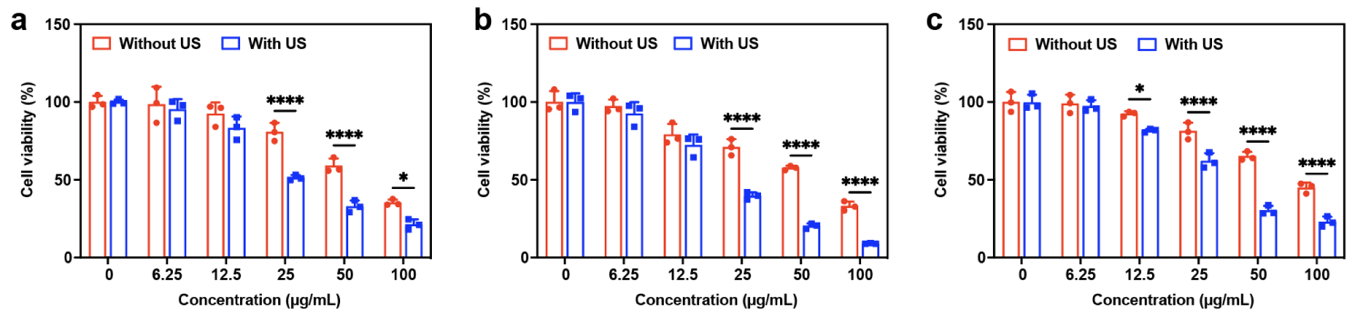


**Figure S15.** Relative cell viability of (a) M0 macrophages, (b) M1 macrophages and (c) M2 macrophages (n = 3). Data are presented as mean values ± SD. **p* < 0.05, *****p* < 0.0001.


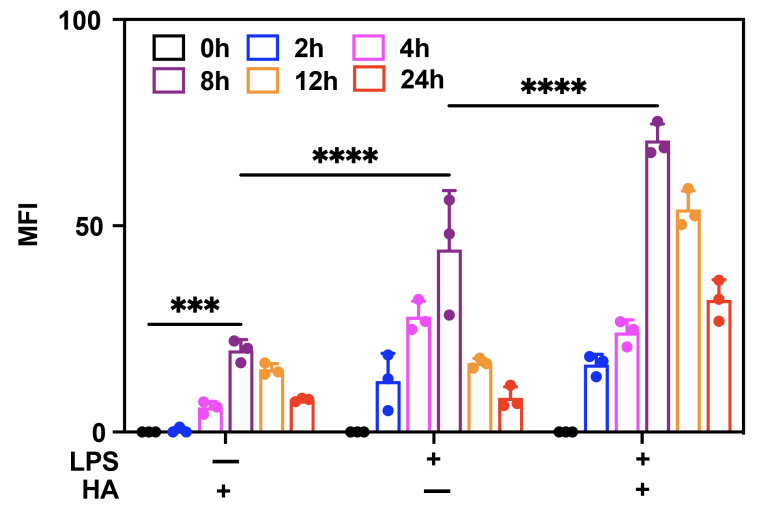


**Figure S16.** Quantitative analysis of Figure 3e (n = 3). Data are presented as mean values ± SD. ****p* < 0.001, *****p* < 0.0001.


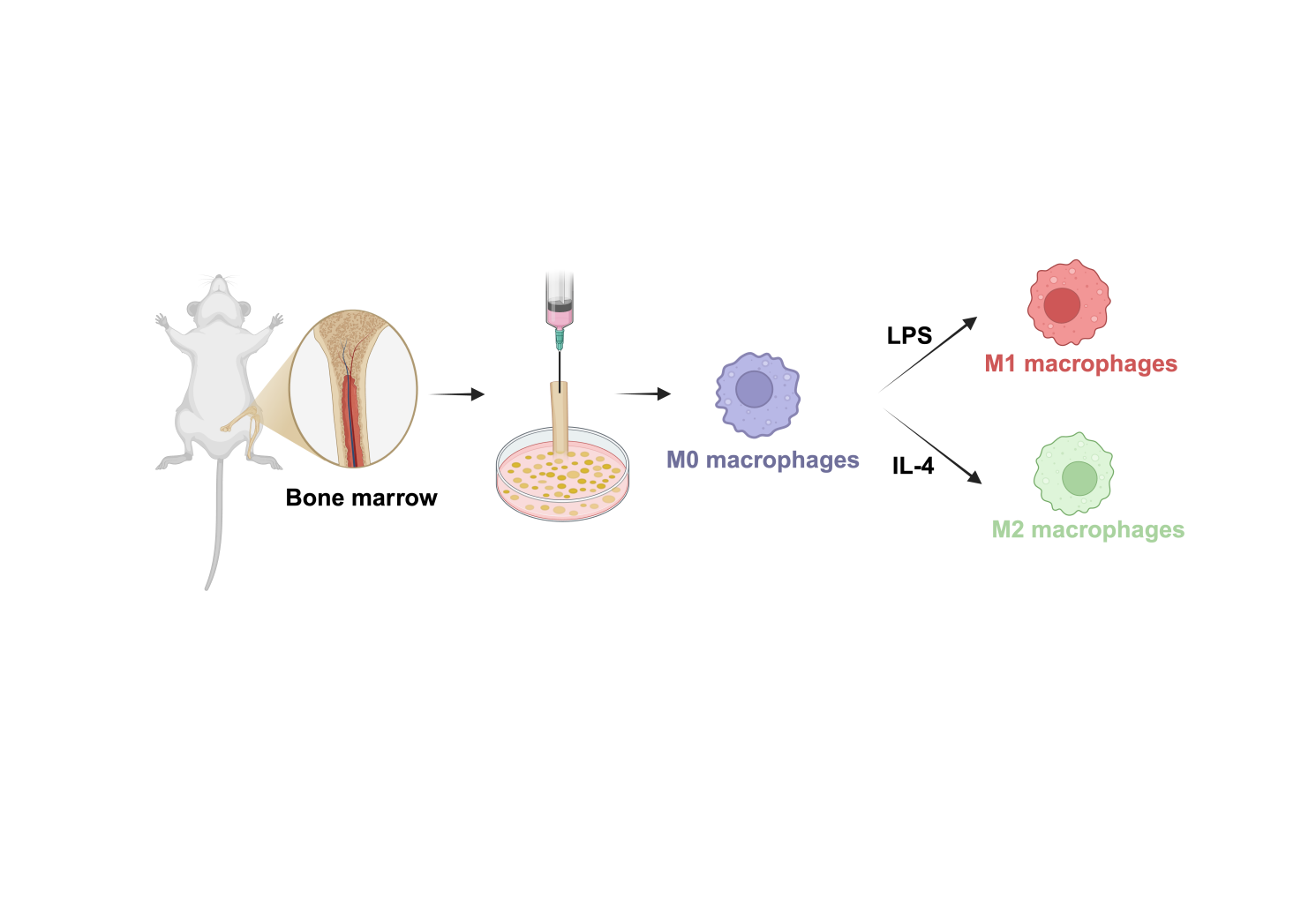


**Figure S17.** Schematic illustration of BMDM extraction and differentiation of macrophages.


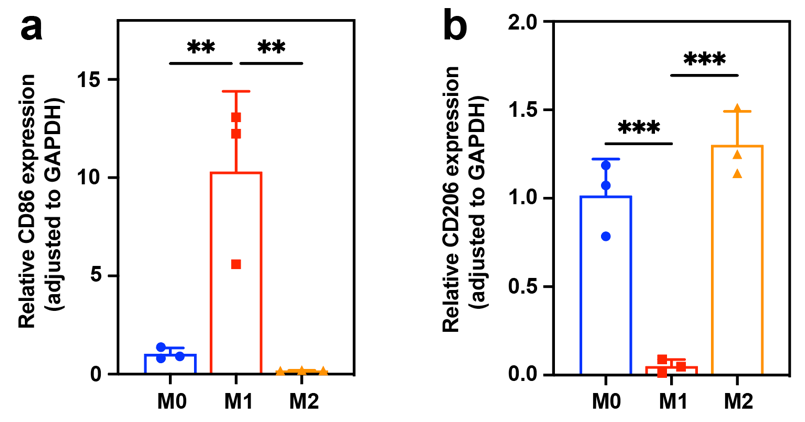


**Figure S18.** Quantitative RT-PCR analysis of RNA levels of (a) CD86, (b) CD206 in different macrophages (n = 3). Data are presented as mean values ± SD. ***p* < 0.01, ****p* < 0.001.


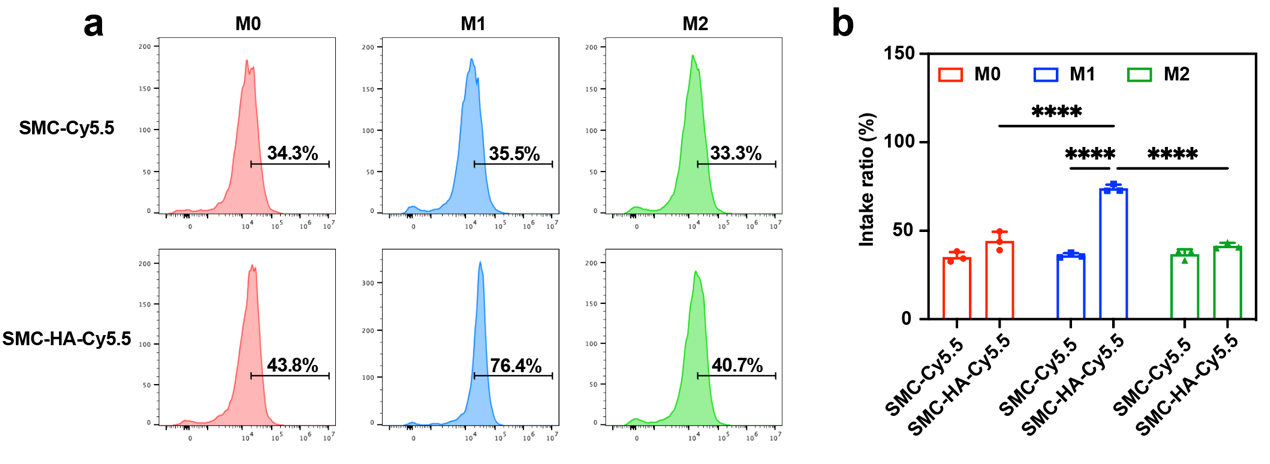


**Figure S19.** (a) Intracellular uptake of SMC and SMC-HA in different macrophages. (b) Quantitative analysis of intake ratio (n = 3). Data are presented as mean values ± SD. *****p* < 0.0001.


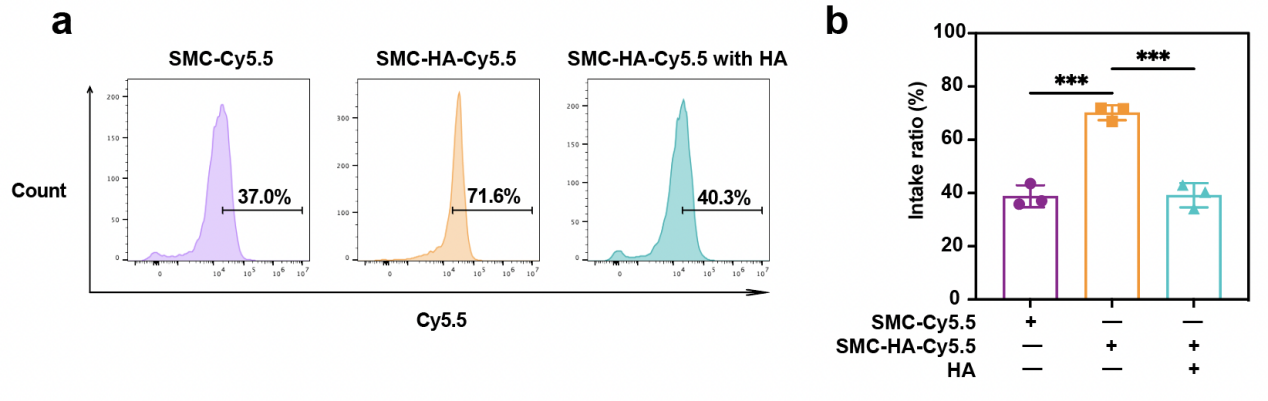


**Figure S20.** (a) Pre-treatment of free HA reduced the targeting effect of SMC-HA in M1 macrophages. (b) Quantitative analysis of intake ratio (n = 3). Data are presented as mean values ± SD. ****p* < 0.001.


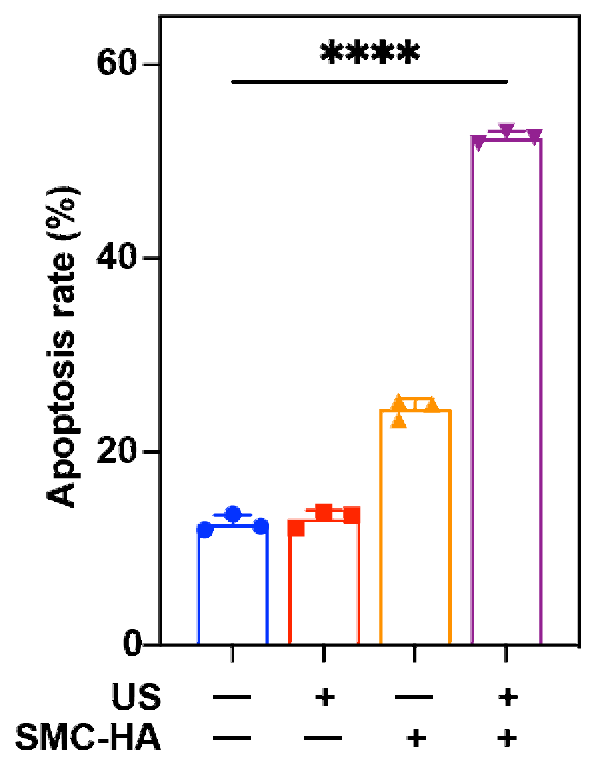


**Figure S21.** Quantitative analysis of Figure 3g (n = 3). Data are presented as mean values ± SD. *****p* < 0.0001.


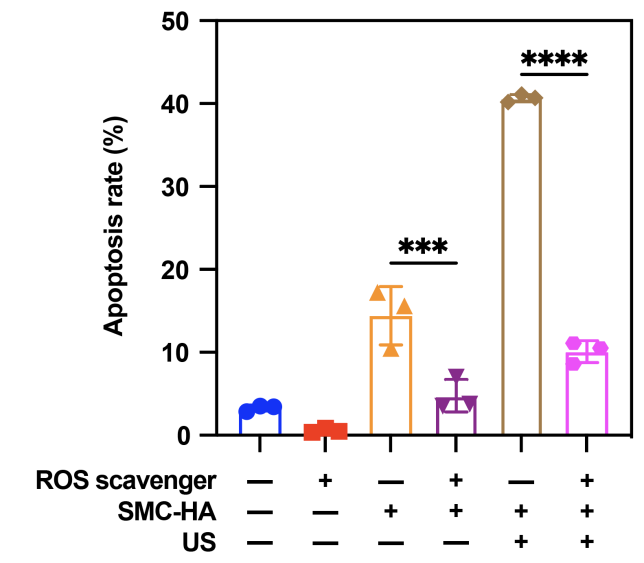


**Figure S22.** Quantitative analysis of Figure 4e (n = 3). Data are presented as mean values ± SD. ****p* < 0.001, *****p* < 0.0001.


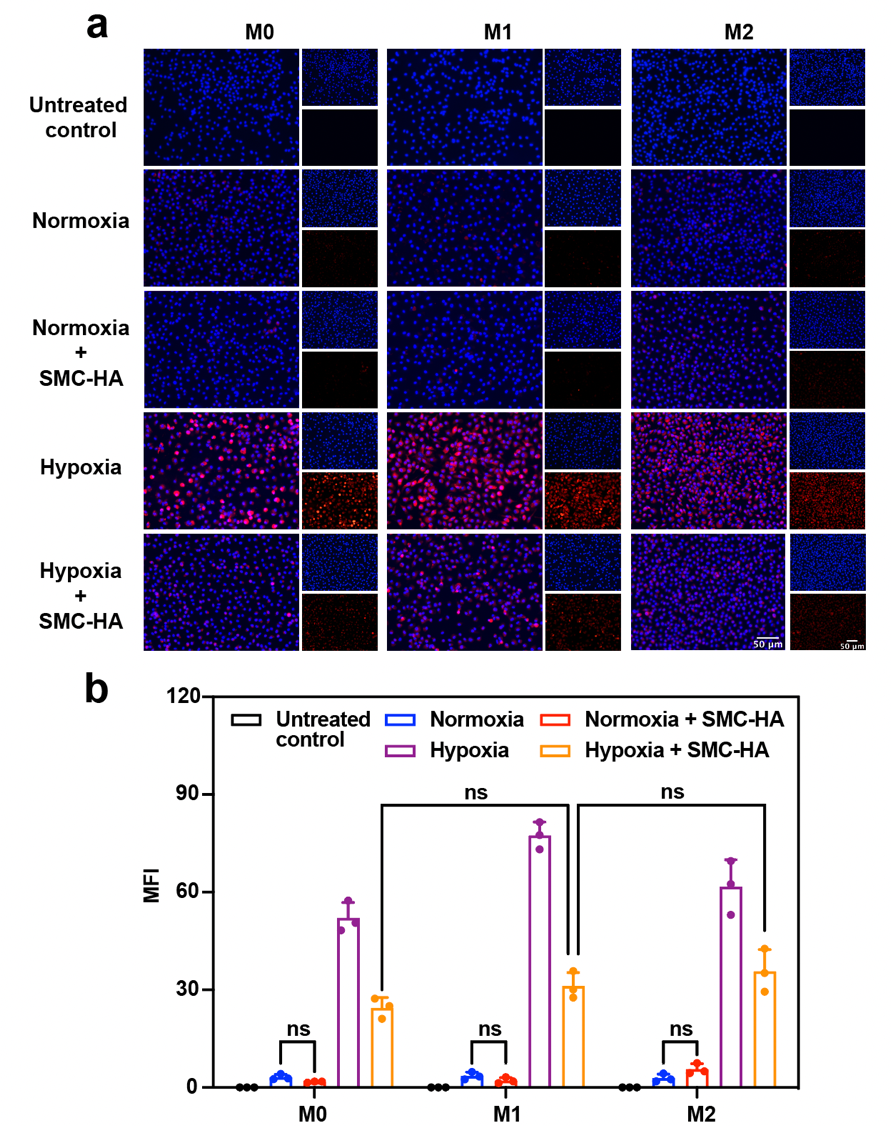


**Figure S23.** (a) Intracellular oxygenation in BMDMs as examined using the oxygen indicator [Ru(dpp)_3_]Cl_2_. Scale bar: 50 μm. (b) Quantitative analysis of the fluorescent intensity (n = 3). Data are presented as mean values ± SD. ns: *p* > 0.05.


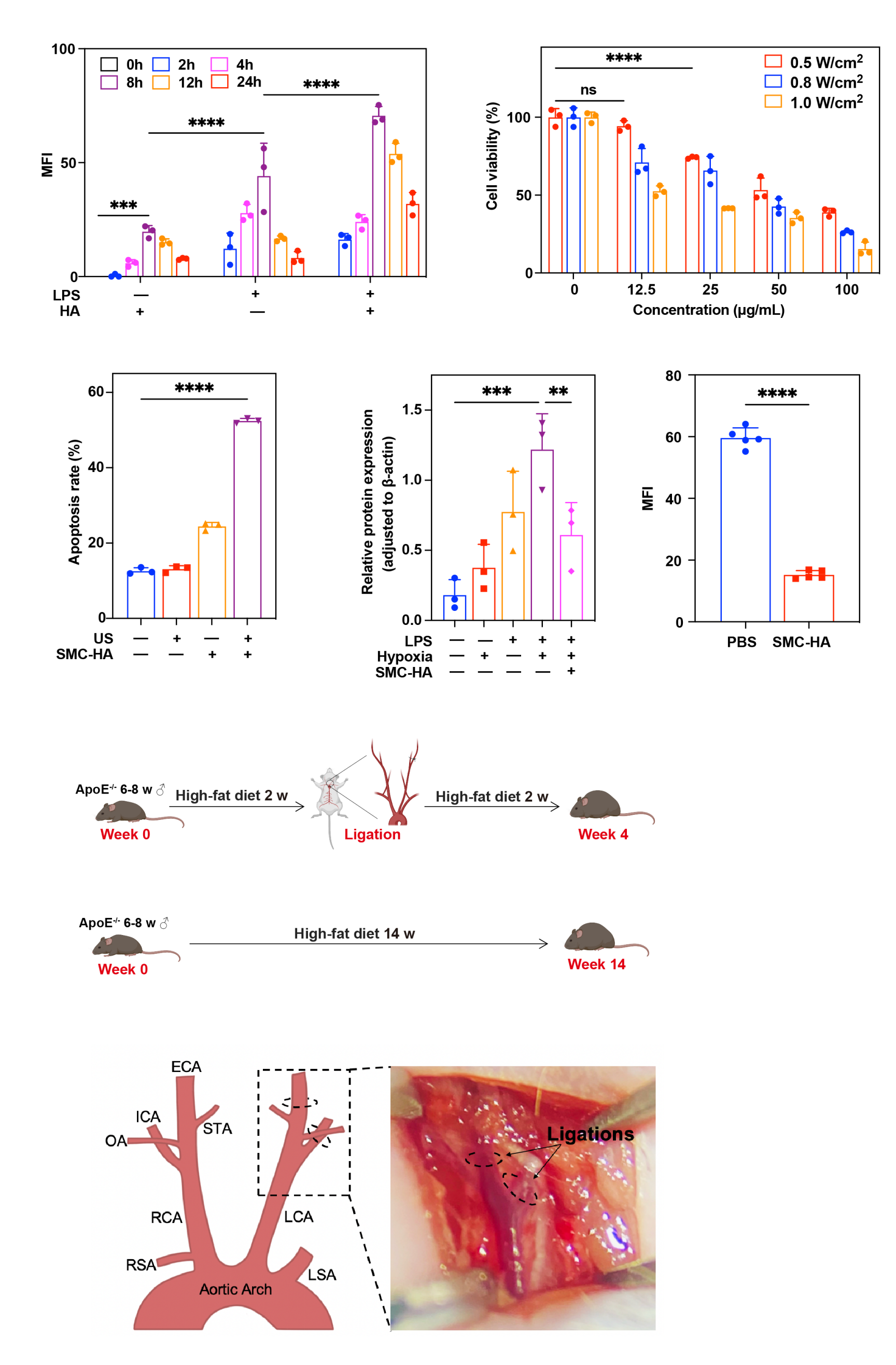


**Figure S24.** Schematic illustration of carotid plaque formation in *ApoE^-/-^* mice induced by 4-week high-fat diet combined with partial left carotid artery ligation.


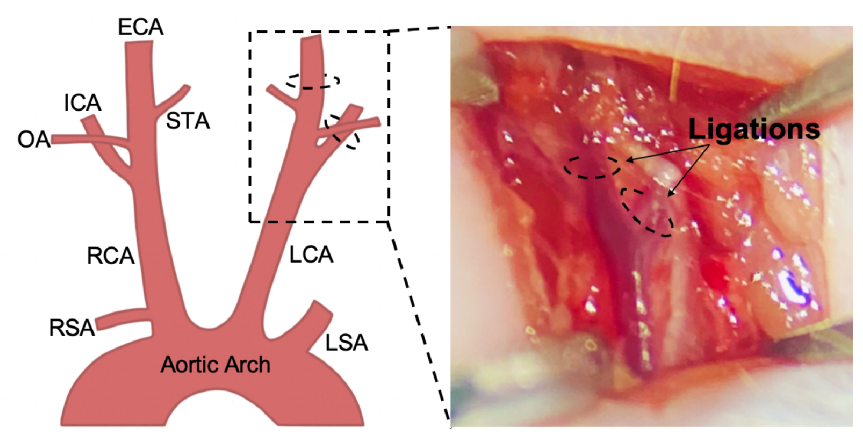


**Figure S25.**Schematic illustration of partial left carotid artery ligation.


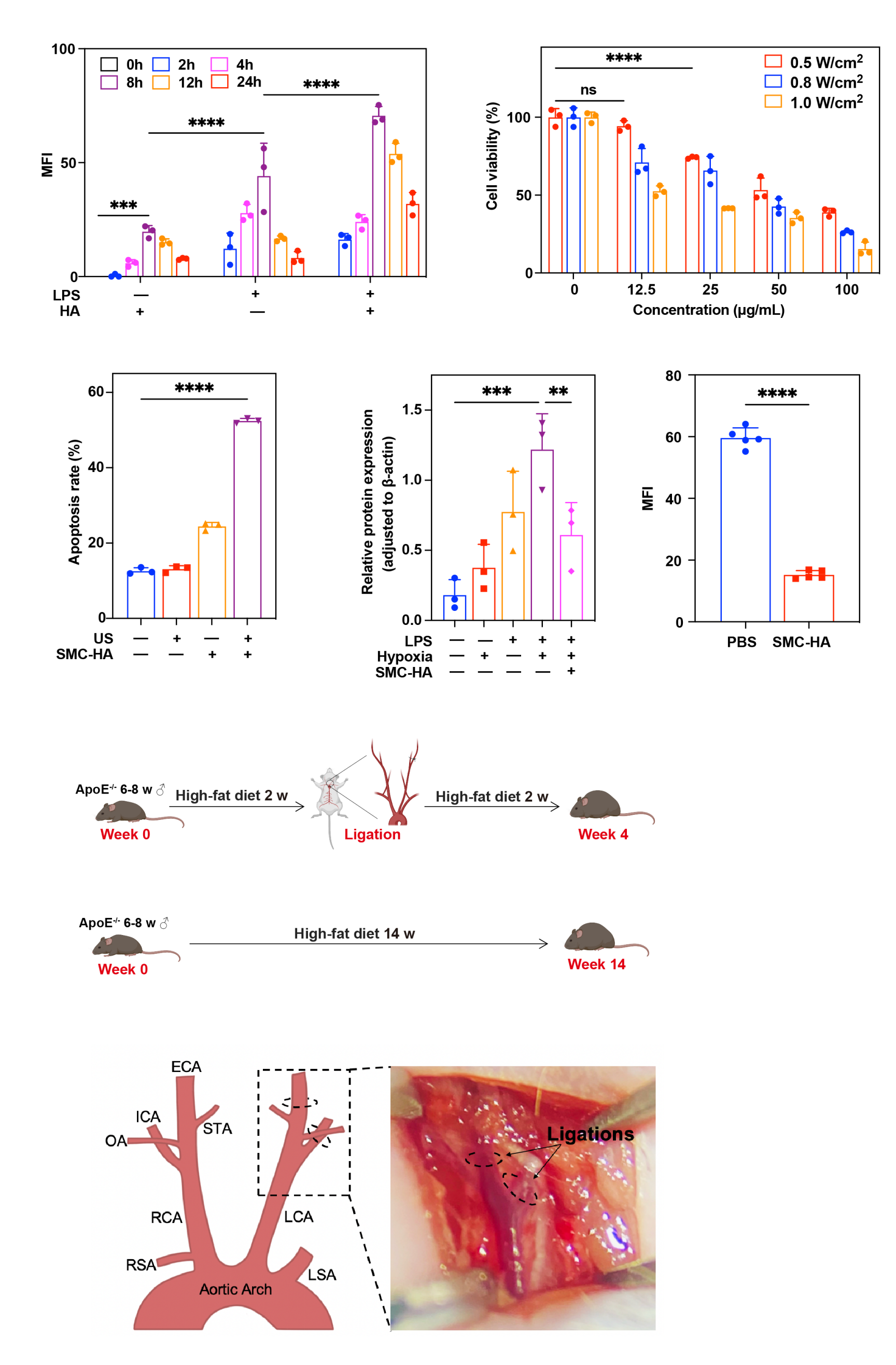


**Figure S26.** Schematic illustration of spontaneous aortic plaque formation in *ApoE^-/-^* mice induced by 14-week of high-fat diet.

**
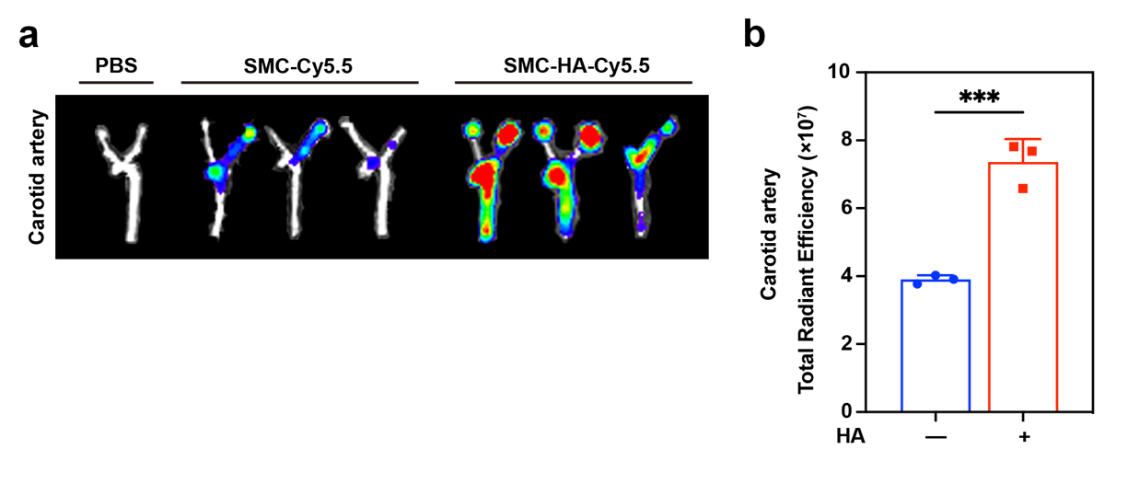
**

**Figure S27.** (a) Fluorescence image of carotid artery and aortic arch from *ApoE^-/-^* mice with 4-week high-fat diet and partial left carotid artery ligation following intravenous injection of PBS and Cy5.5-labeled nanoparticles. (b) Quantitative analysis of the fluorescent intensity (n = 3). Data are presented as mean values ± SD. ****p* < 0.001.


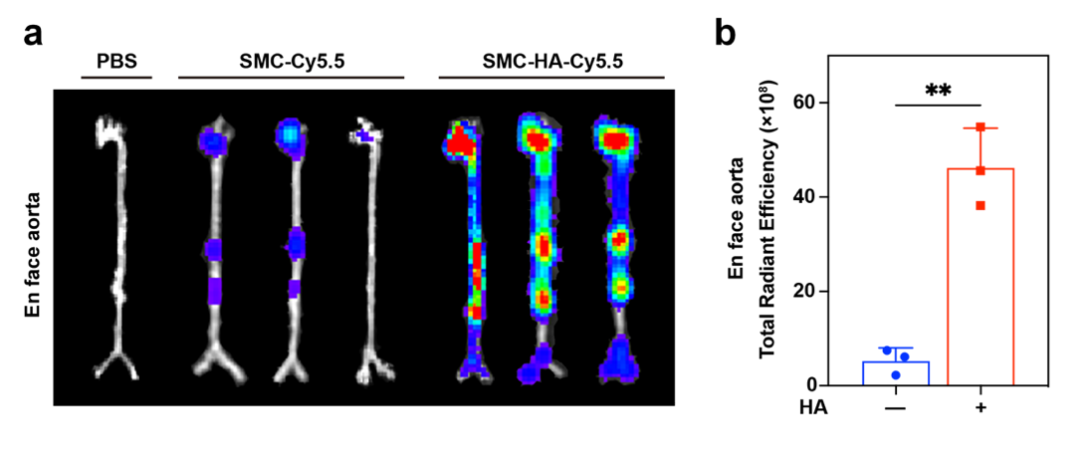


**Figure S28.** Fluorescence image of aortas from *ApoE^-/-^* mice with 14-week high-fat diet following intravenous injection of PBS and Cy5.5-labeled nanoparticles. Quantitative analysis of the fluorescent intensity (n = 3). Data are presented as mean values ± SD. ***p* < 0.01.


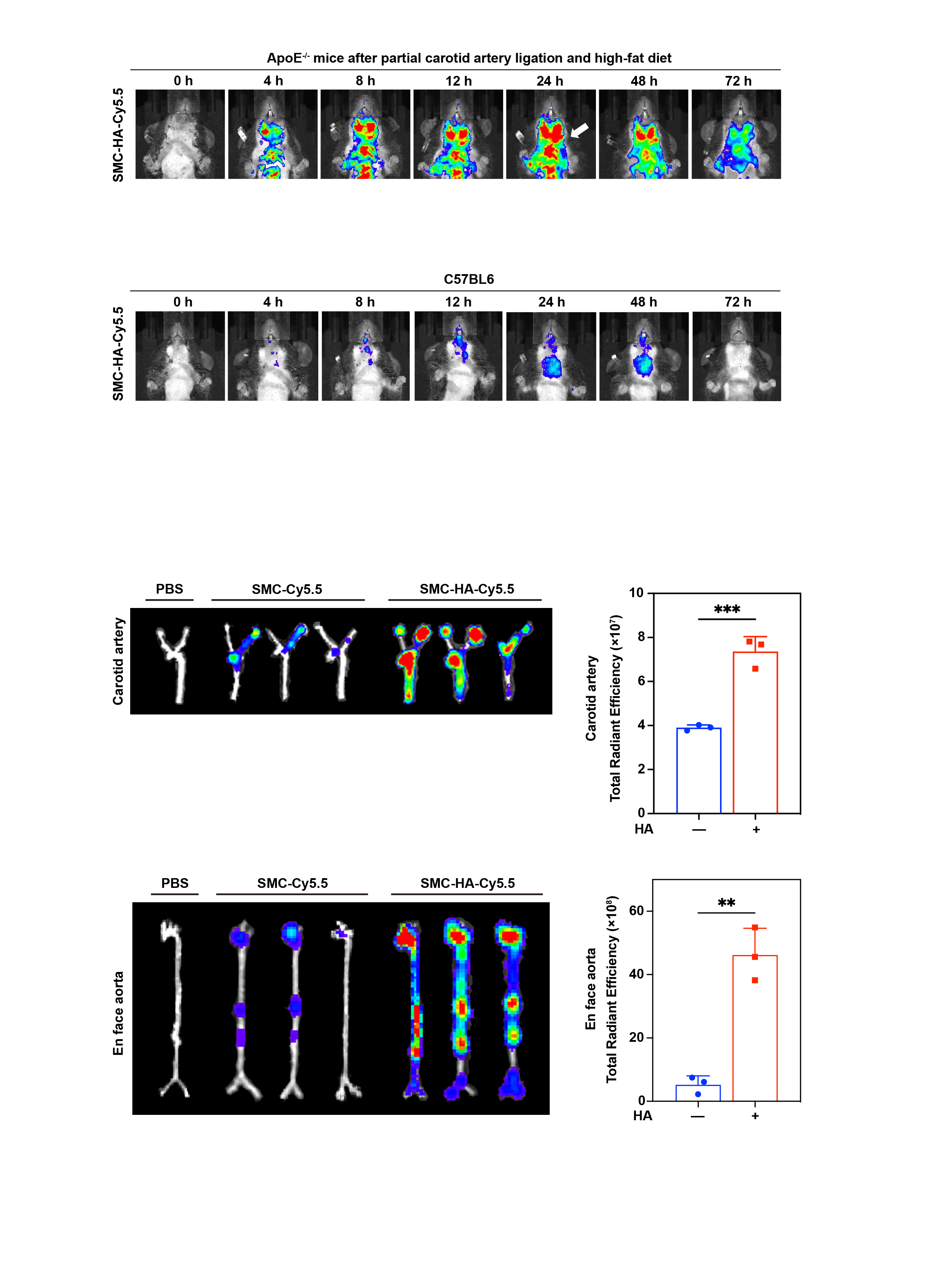


**Figure S29.** Schematic illustration of in vivo imaging of SMC-HA in *ApoE^-/-^* mice with 4-week

high-fat diet and partial left carotid artery ligation over time.


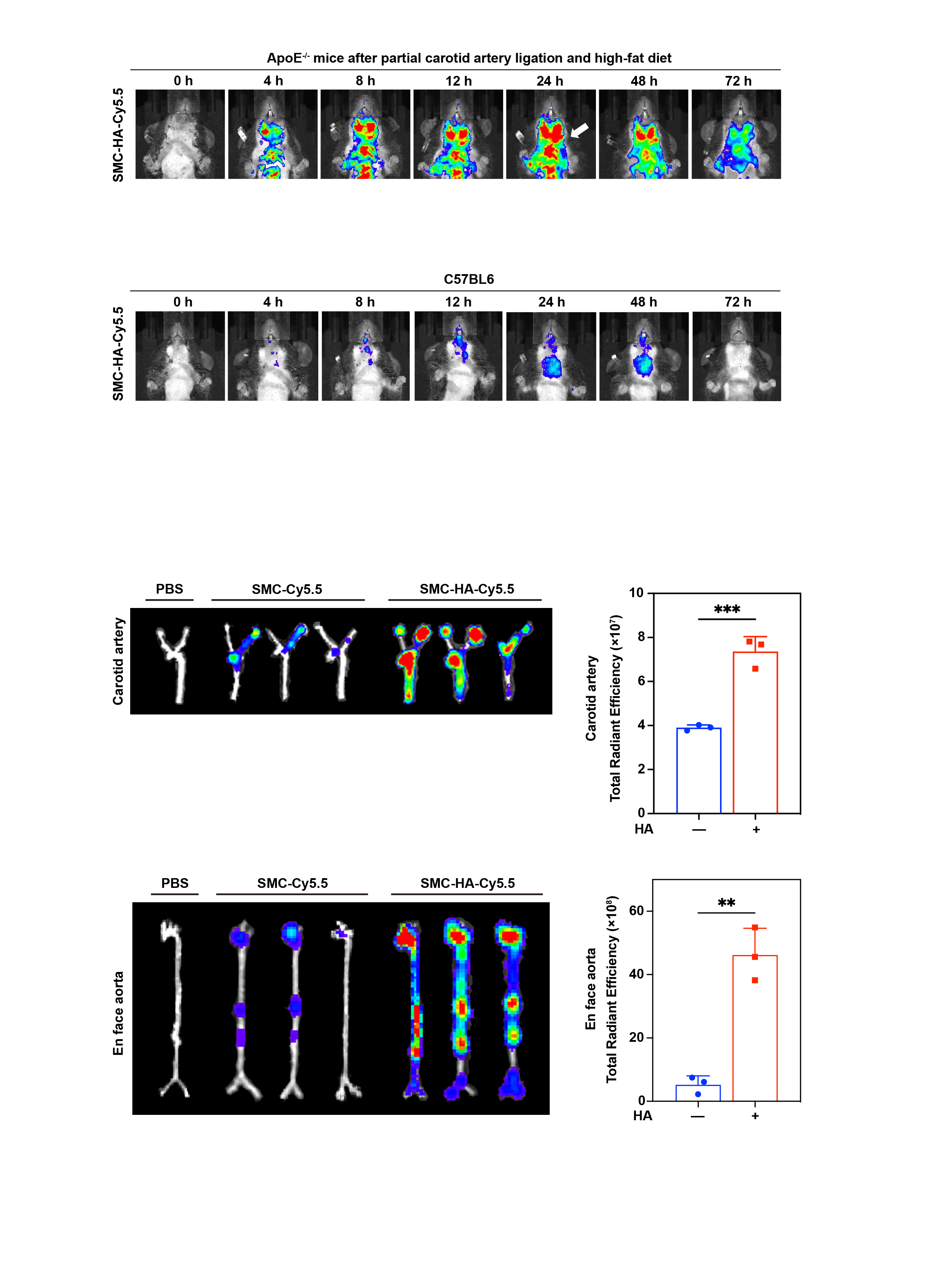
**Figure S30.** Schematic illustration of *in vivo* imaging of SMC-HA in C57BL6 mice over time.


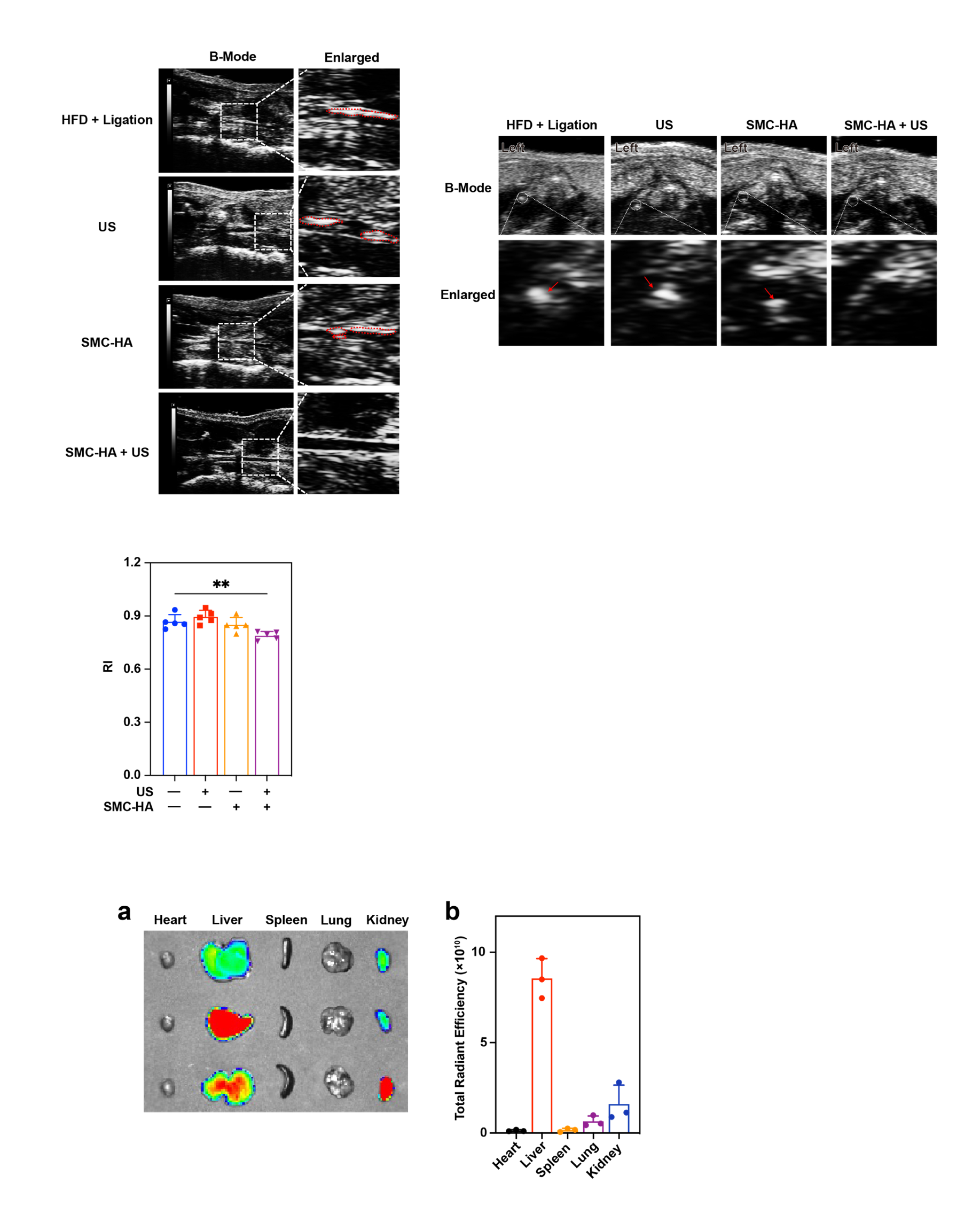


**Figure S31.** B-mode ultrasound visualized left common carotid artery morphology (long axis). The plaques are circled by red dotted lines.


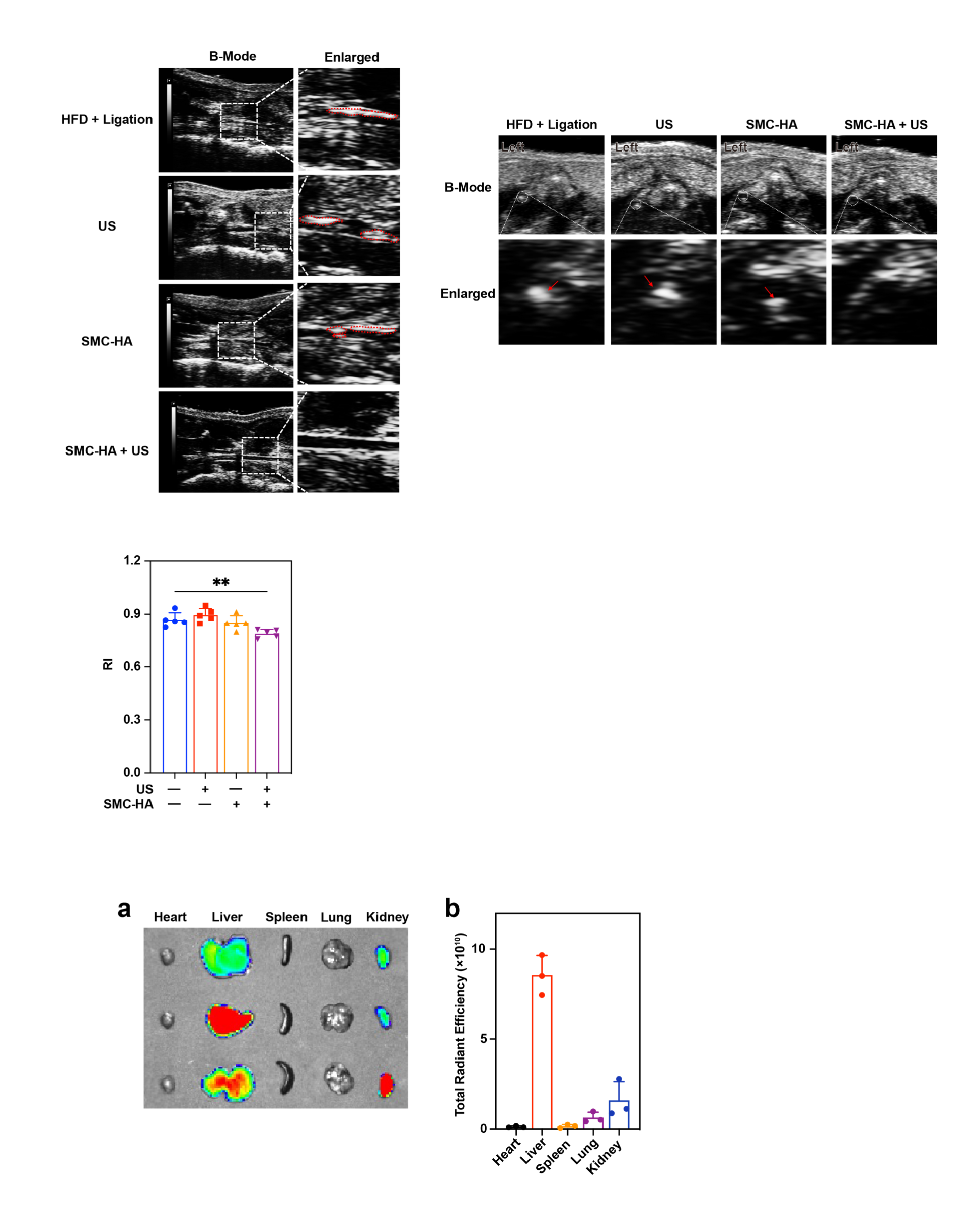


**Figure S32.** B-mode images of transverse section views of atherosclerotic plaques in left common carotid artery (short axis).


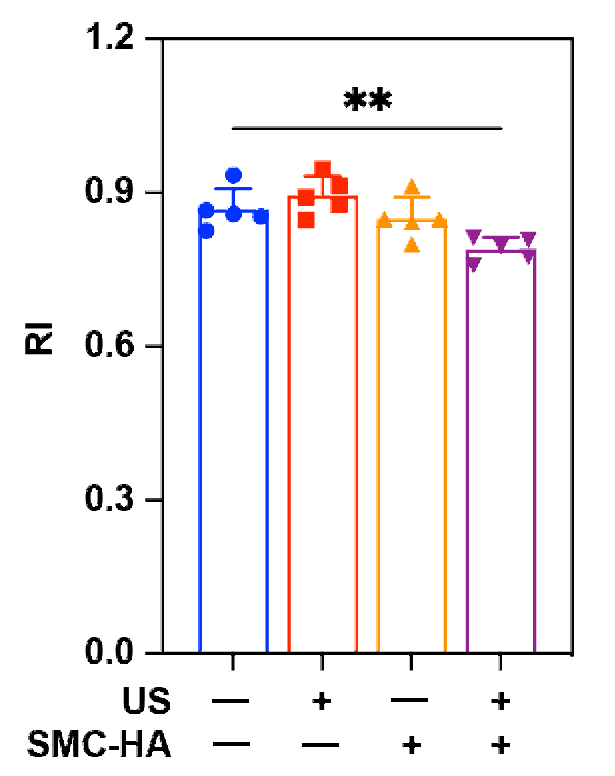


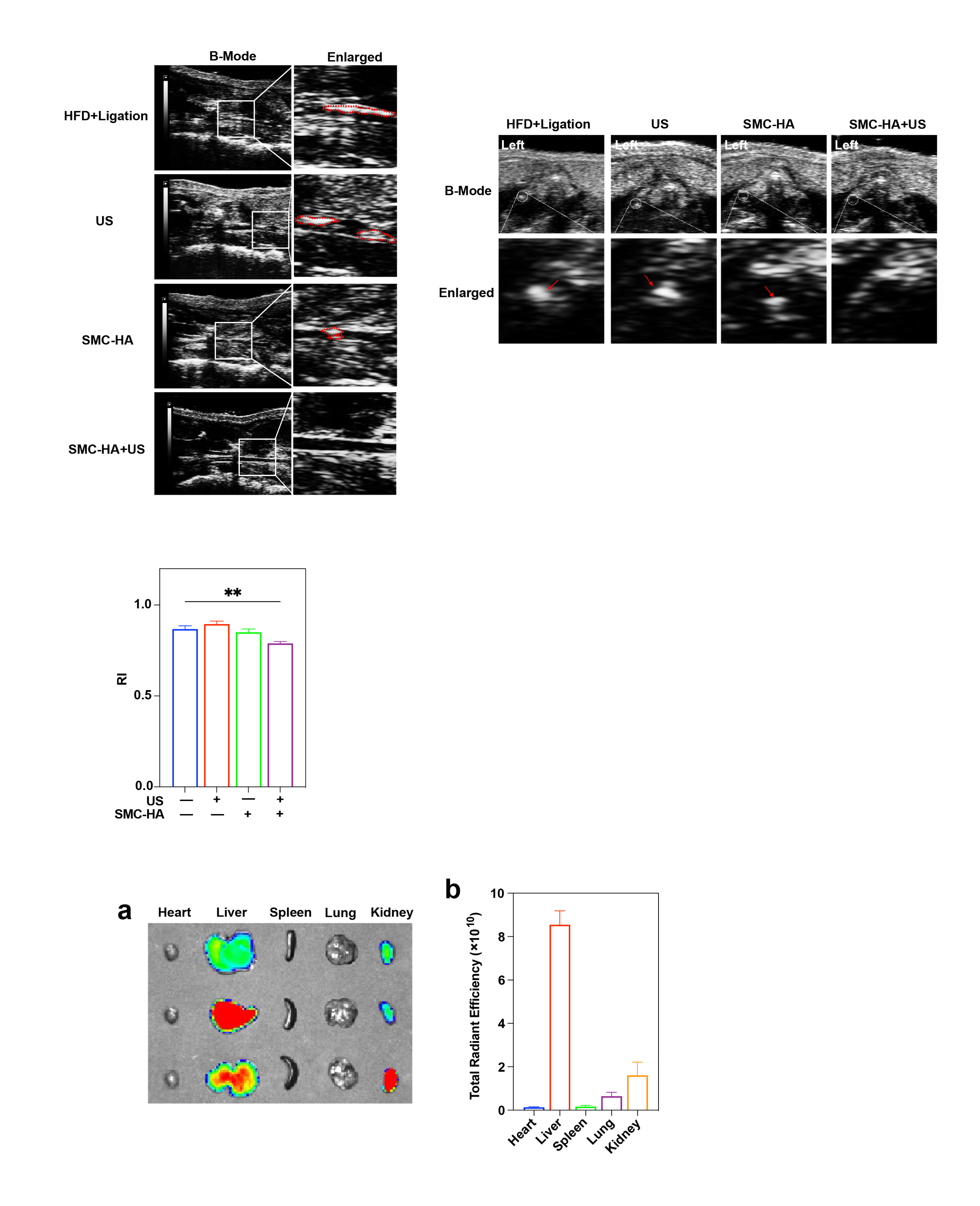
**Figure S33.** Statistical analysis of the RI value in Figure 5e (n = 5). RI: resistance index. Data are presented as mean values ± SD. ***p* < 0.01.


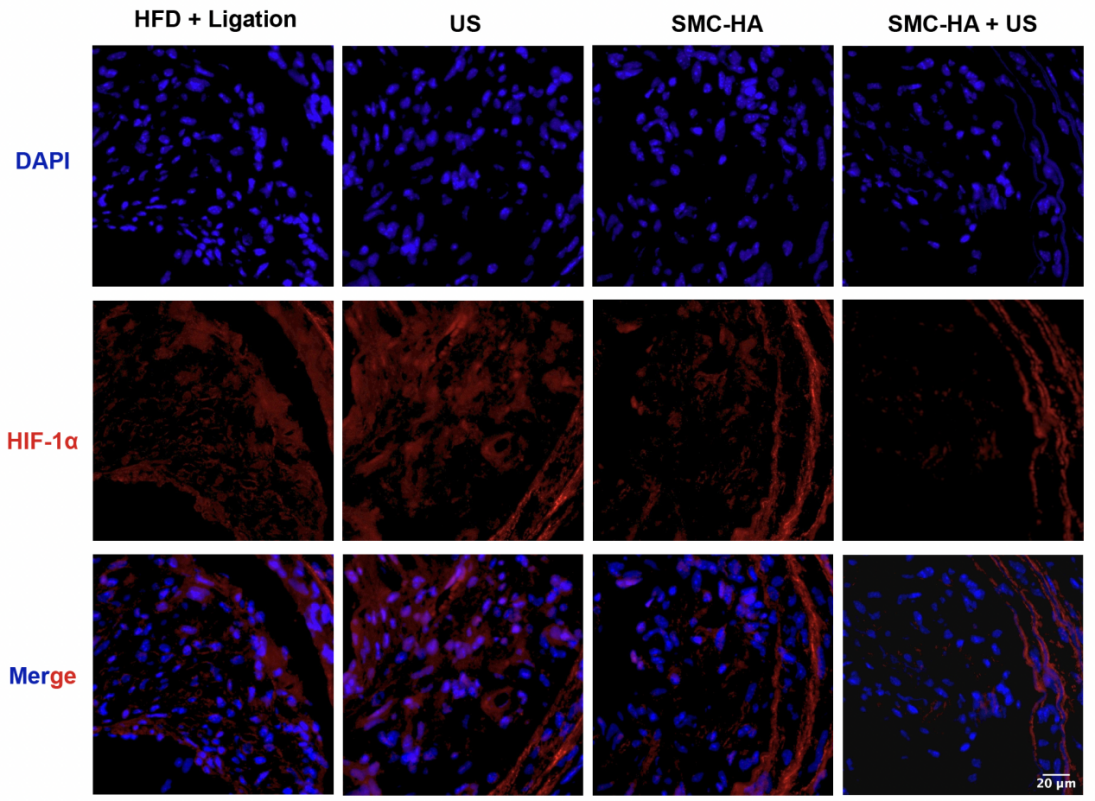


**Figure S34.** Immunofluorescence of HIF-1α in LCCA transverse sections isolated from *ApoE^-/-^* mice with partial left carotid artery ligation and 4-week high-fat diet feeding. Scale bar: 20 μm.


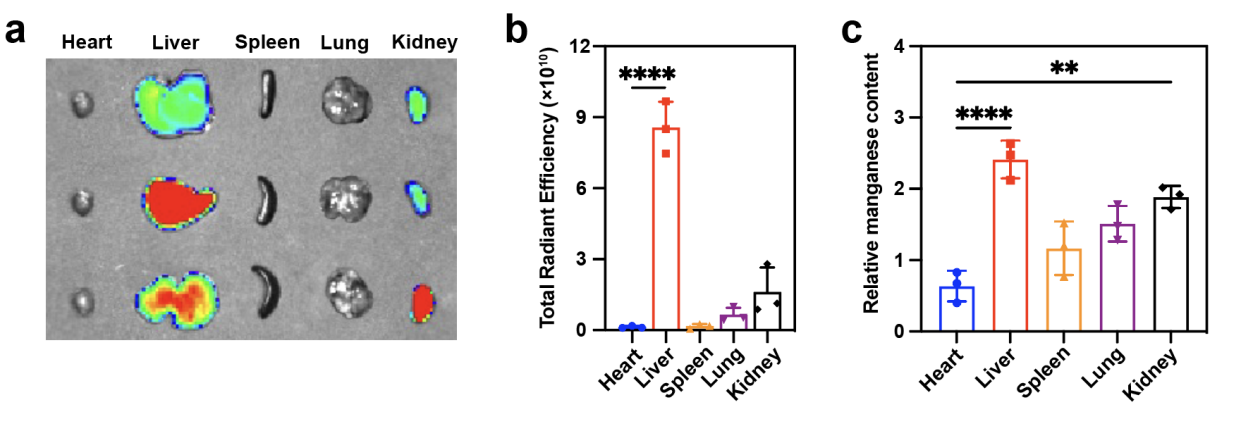


**Figure S35.** (a) Fluorescence image of major organs from *ApoE^-/-^* mice with 4-week high-fat diet and partial left carotid artery ligation following intravenous injection of SMC-HA-Cy5.5. (b) Quantitative analysis of the fluorescent intensity (n = 3). (c) Ex vivo ICP-MS assay results of manganese ion content in different organs at 24 h after injection (n=3). Data are presented as mean values ± SD. ***p* < 0.01, *****p* < 0.0001.

**Table S1.** The primer sequences used for real-time quantitative PCR.

| **Primers** | **Forward/Reverse** | **Sequence (5' → 3')** |
| --- | --- | --- |
| GAPDH | Forward | AGGTCGGTGTGAACGGATTTG |
|  | Reverse | TGTAGACCATGTAGTTGAGGTCA |
| IL-1β | Forward | GCAACTGTTCCTGAACTCAACT |
|  | Reverse | ATCTTTTGGGGTCCGTCAACT |
| TNF-α | Forward | CCCTCACACTCAGATCATCTTCT |
|  | Reverse | GCTACGACGTGGGCTACAG |
| CD44 | Forward | TCGATTTGAATGTAACCTGCCG |
|  | Reverse | CAGTCCGGGAGATACTGTAGC |
| CD86 | Forward | TGTTTCCGTGGAGACGCAAG |
|  | Reverse | TTGAGCCTTTGTAAATGGGCA |
| CD206 | Forward | CTCTGTTCAGCTATTGGACGC |
|  | Reverse | CGGAATTTCTGGGATTCAGCTTC |
